# Hippocampal and cortical activities reflect early hyperexcitability in an Alzheimer's mouse model

**DOI:** 10.1093/braincomms/fcaf443

**Published:** 2025-11-12

**Authors:** Marina Diachenko, Georgii Krivoshein, Arn M J M van den Maagdenberg, Huibert D Mansvelder, Ronald E van Kesteren, Else A Tolner, Klaus Linkenkaer-Hansen

**Affiliations:** Department of Integrative Neurophysiology, Center for Neurogenomics and Cognitive Research, Amsterdam Neuroscience, Vrije Universiteit Amsterdam, Amsterdam 1081 HV, The Netherlands; Department of Human Genetics, Leiden University Medical Center, Leiden 2333 ZC, The Netherlands; Department of Neurology, Leiden University Medical Center, Leiden 2333 ZC, The Netherlands; Department of Human Genetics, Leiden University Medical Center, Leiden 2333 ZC, The Netherlands; Department of Neurology, Leiden University Medical Center, Leiden 2333 ZC, The Netherlands; Department of Integrative Neurophysiology, Center for Neurogenomics and Cognitive Research, Amsterdam Neuroscience, Vrije Universiteit Amsterdam, Amsterdam 1081 HV, The Netherlands; Department of Molecular and Cellular Neurobiology, Center for Neurogenomics and Cognitive Research, Vrije Universiteit Amsterdam, Amsterdam 1081 HV, The Netherlands; Department of Human Genetics, Leiden University Medical Center, Leiden 2333 ZC, The Netherlands; Department of Neurology, Leiden University Medical Center, Leiden 2333 ZC, The Netherlands; Department of Integrative Neurophysiology, Center for Neurogenomics and Cognitive Research, Amsterdam Neuroscience, Vrije Universiteit Amsterdam, Amsterdam 1081 HV, The Netherlands

**Keywords:** Alzheimer's disease, excitation–inhibition balance, functional E/I ratio, neuronal oscillations, local field potential

## Abstract

Early stages of Alzheimer's disease are marked by brain hyperexcitability, evidenced by subclinical epileptiform features suggesting an excitation–inhibition imbalance. Clinically translatable biomarkers for early detection of excitation–inhibition changes at the network level, however, are lacking. We investigated the functional excitation–inhibition ratio, theta–gamma phase–amplitude coupling and epileptiform features in hippocampal and cortical local field potentials recorded weekly in freely behaving male APPswe/PS1dE9 (APP/PS1) mice (*n* = 10) and wild-type controls (*n* = 10) between 3 and up to and including 11 months of age. APP/PS1 mice exhibited a shift towards increased excitation, reflected in the elevated functional excitation–inhibition ratio emerging most prominently in the hippocampus at 6 months. Additionally, elevated population spiking activity and age-related impairments in theta–gamma phase–amplitude coupling were observed in the local field potentials of APP/PS1 mice in both the hippocampus and the cortex. Importantly, the functional excitation–inhibition ratio correlated positively with elevated population spiking activity in both brain regions in APP/PS1 mice. Our findings highlight the functional excitation–inhibition ratio as a promising biomarker of hippocampal and cortical network disinhibition and hyperexcitability in APP/PS1 mice, with potential value as an early disease marker in Alzheimer's disease.

## Teaser

Diachenko *et al*. report a quantitative electroencephalography-based marker for tracking early-stage hyperexcitability in Alzheimer's disease. In an Alzheimer's mouse model, longitudinal cortical and hippocampal recordings revealed early-stage frequency-specific shifts towards excitatory dominance. They also found impaired theta–gamma phase–amplitude coupling and elevated population spiking over the course of disease progression.

## Graphical Abstract

**Graphical Abstract:**
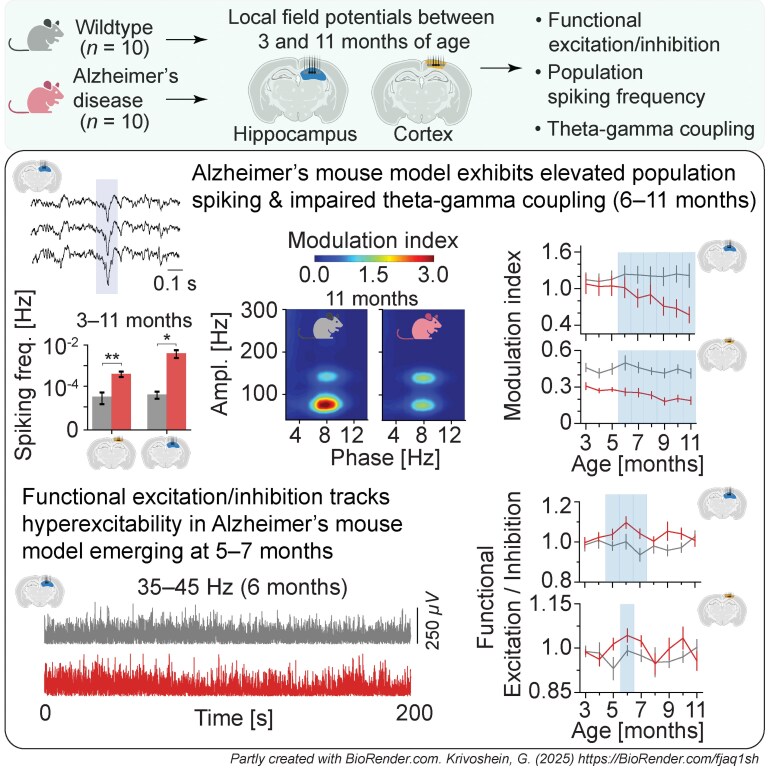

## Introduction

Alzheimer's disease is a progressive neurodegenerative disorder characterized by pathological changes, including amyloid-β (Aβ) plaque accumulation in the brain, that begin decades before hallmark clinical symptoms such as memory loss emerge.^1-3^ This long ‘preclinical’ stage, when people remain cognitively normal despite an already ongoing disease process in their brains, presents an excellent window of opportunity to identify at-risk individuals and initiate therapeutic interventions, as some of the early pathological changes may still be reversible.^3-5^ In this regard, a widely recognized early hallmark of disease onset in Alzheimer's disease patients and mouse models is a disturbed brain network organization, particularly an imbalance of neuronal excitation and inhibition (E/I).^6-8^ Early symptoms of hyperexcitability, which includes subclinical epileptiform activity, have been observed in both Alzheimer's disease patients^9-11^ and animal models^12,13^ and have been indicated to potentially contribute to early cognitive impairment.^11,14,15^

Although a prominent indicator of network hyperexcitability, subclinical epileptiform activity is not suitable as a clinical biomarker. Neuronal circuits can be in a primed hyperexcitable state—characterized by increased neuronal firing or a reduced threshold for excitation—without or prior to overt seizures or epileptiform discharges being detectable by electroencephalography (EEG) or local field potential (LFP), as indicated also from observations in preclinical Alzheimer's disease models.^16,17^ Notably, subcortical epileptiform features often remain undetected by scalp EEG.^18^ Subclinical (i.e. non-convulsive) epileptiform discharges as detected from scalp EEG appear in only about half of Alzheimer's disease patients,^9,10,19^ and their prevalence is even lower during preclinical Alzheimer's disease and mild cognitive impairment (MCI) stages.^11^ Moreover, these discharges are generally infrequent, with about two to four events per hour on average, and occur predominantly during sleep.^10,19^ This pattern complicates their detection on routine EEG and may demand serial or long-term monitoring. Healthy individuals can also show subclinical epileptiform activity, albeit less often and without known clinical consequences. In addition, true epileptiform discharges can be mistaken for artefacts or normal variants.^20^ Therefore, an ultimate challenge in this context is to develop a quantifiable biomarker based on EEG recordings of brain activity that reflects the extent of E/I imbalance in Alzheimer's disease patients. Such a biomarker could help identify people at risk of developing Alzheimer's disease and be used to monitor disease progression and assess cognitive decline in an objective manner. Despite growing evidence supporting the neuronal network imbalance hypothesis in Alzheimer's disease, detecting such network changes in presymptomatic or early symptomatic Alzheimer's disease patients remains challenging.

Building on a computational model of critical brain dynamics, we recently developed a network-level measure of the functional E/I (fE/I) ratio to detect E/I changes from neuronal network oscillations recorded with human EEG.^21,22^ In this framework, E/I balance is conceptualized as an emergent property of critical dynamics within networks poised between order and disorder. The fE/I measure is sensitive to simulated changes in both network connectivity and synaptic function and responds to pharmacological manipulation in humans.^21^ Under healthy conditions, fE/I typically hovers around 1––implying a globally balanced E/I ratio––but it becomes imbalanced in various brain pathologies,^21,23-25^ with more excited or inhibited networks showing an fE/I > 1 or fE/I < 1, respectively.^21^ In early-stage Alzheimer's disease patients, fE/I findings remain inconsistent,^26,27^ underscoring the difficulty of using this measure to elucidate an E/I imbalance in Alzheimer's disease. Thus far, fE/I has primarily been applied in clinically relevant neurophysiological signals in humans, hampering its mechanistic interpretation; there is only one preclinical application, namely, in a mouse model of autism spectrum disorder.^28^

In this study, we explored whether fE/I can serve as a biomarker of network E/I imbalance in the early stages of disease development in APPswe/PSEN1de9 mice (hereafter referred to as APP/PS1 mice). The APP/PS1 model is a well-established mouse model of Alzheimer's disease replicating key pathological features and cognitive impairments of clinical Alzheimer's disease,^29,30^ in which early network changes include aberrant hippocampal inhibitory neuronal activity and epileptiform activities associating with Aβ pathogenesis.^12,31-33^ To assess E/I network features, we computed fE/I from LFP signals recorded in the hippocampus (HC; CA1 area) and cortex of freely behaving APP/PS1 mice during quiet wakefulness, collecting data weekly over several months. Given the limited prior research on applying the fE/I algorithm in mice, we first confirmed that, similar to human EEG and magnetoencephalography (MEG) data, fE/I can be reliably derived from mouse LFP signals to infer the E/I ratio of narrow-band oscillatory activity at the single-electrode level. Next, we assessed fE/I in narrow frequency ranges spanning the 1–150 Hz spectrum and compared age-related fE/I profiles in CA1 and the parietal cortex (PTC) between APP/PS1 and wild-type (WT) mice. To further explore E/I network features in APP/PS1 mice, we incorporated theta–gamma (θ–γ) phase–amplitude coupling (PAC), an E/I metric that strongly relies on hippocampal–cortical inhibitory gamma-aminobutyric acid (GABA)–mediated (GABAergic) network activity.^34,35^ This measure supports memory^36,37^ and is impaired in Alzheimer's disease patients^38^ and animal models.^39-41^ Finally, to evaluate fE/I as a potential clinical marker of hippocampal–cortical E/I changes in Alzheimer's disease, fE/I features were correlated with θ–γ PAC and epileptiform activity.

## Materials and methods

### Data collection and preprocessing

**Figure 1:**
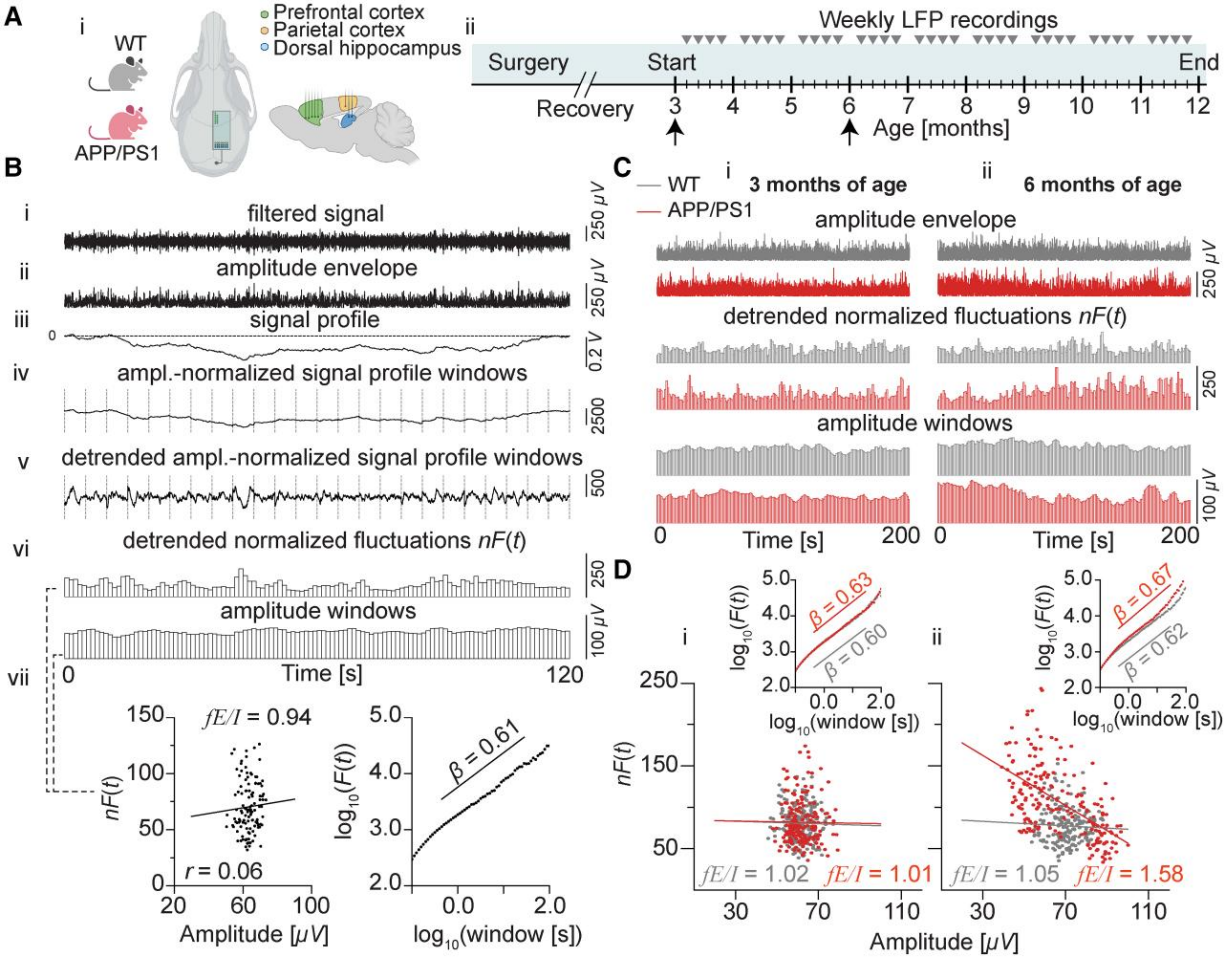
**fE/I applied to a mouse LFP signal to infer hippocampal and cortical E/I ratios in APP/PS1 and WT mice.** (**A**) The placement of LFP electrodes in the prefrontal cortex, parietal cortex and dorsal hippocampus of APP/PS1 and WT mice (i). Experimental timeline for hippocampal and cortical LFP surgery and longitudinal recordings starting from the age of 3 and ending when a mouse turned 12 months; arrow heads indicating the weekly LFP recordings and arrows indicating the time points used for the example analyses in **C** below (ii). (**B**) Analysis steps of the fE/I ratio algorithm. First, a LFP signal is filtered in the desired low-gamma frequency range (35–45 Hz) (i), and the amplitude envelope is extracted (ii). Next, a signal profile is created (iii) that is divided into 5 s windows with 80% overlap (iv) (for illustration purposes, shown without overlap). Each window is normalized by the mean of its amplitude envelope. The normalized windows are then detrended (v), and the root mean square fluctuation of the detrended amplitude-normalized signal profile windows gives the normalized fluctuation function, *nF*(*t*) (vi). The fE/I value is obtained as 1 minus the Pearson correlation between the windowed amplitude (*x*-axis) and the normalized fluctuation values *nF*(*t*) (*y*-axis) (vii, left). Each dot represents a 5 s window. Beforehand, LRTCs are estimated via the DFA exponent, *β*; fE/I cannot be computed if *β* < 0.6 (vii, right). Each dot represents the average fluctuation (root mean square deviation) of the signal for a given window size. (**C**) Example computation for a LFP signal from a hippocampal electrode in the pyramidal CA1 layer from a mouse in the quiet awake state. The signal was filtered in the range of 35–45 Hz for a WT mouse and an APP/PS1 mouse at 3 (i) and 6 (ii) months of age, respectively (time points indicated by vertical arrows in **A**, ii). (**D**) fE/I ratios computed for the two mice at 3 (i) and 6 (ii) months of age as 1 minus the Pearson correlation between the windowed amplitude (*x*-axis) and the normalized fluctuation values *nF*(*t*) (*y*-axis). Each dot represents a 5 s window. The insets show that amplitude fluctuations of hippocampal network activity measured from LFP in both mice exhibit LRTC as indicated by the DFA component *β* > 0.6 at both 3 (i) and 6 (ii) months. The DFA exponent was estimated as the slope of the mean fluctuation as a function of window size, *F*(*t*), fitted in the range of 0.4–30 s in log–log coordinates. Each dot represents the average fluctuation of the signal for a given window size. WT, wild-type; APP/PS1, APPswe/PSEN1de9; DFA, detrended fluctuation analysis; LRTCs, long-range temporal correlations; fE/I, functional excitation–inhibition; LFP, local field potential. The depicted top view of the mouse skull with electrode configuration and the sagittal brain view in **A** were created with BioRender.com (Krivoshein, G. (2025); https://BioRender.com/4i8hboa).

In this study, published LFP data were used that had been recorded from male APP/PS1-PV-Cre mice.^42^ These mice are indistinguishable from APP/PS1 mice in the absence of Cre-dependent intervention.^31^ In brief, LFP recordings of 10–15 min were collected weekly during the light phase (2–6 h after the lights were switched on), starting when the mice were 3 months of age (*n* = 14 WT; *n* = 16 APP/PS1) and continuing through 11 months of age (*n* = 10 WT; *n* = 10 APP/PS1) (Fig. 1A). Over the course of the study, six APP/PS1 mice died, and four WT mice had irrecoverable signal loss. In our study, only the surviving APP/PS1 mice and WT mice without signal loss were used. For details on animals as well as signal acquisition and cleaning, see van Heusden *et al*.^42^

For a detailed description of the used custom-made LFP electrode arrays, surgery and channel selection, see van Heusden *et al*.^42^ In short, nine electrodes were located in the prefrontal cortex (PFC). Seven electrodes were located in the PTC, of which four were *post hoc* selected for analyses. If one of the electrodes did not produce a clean signal or the number of included electrodes per brain region dropped below four, an adjacent electrode was selected. In the HC, a total of 14 electrodes were present, which were grouped based on their location relative to the pyramidal layer, i.e. supra-pyramidal, pyramidal or infra-pyramidal. The location of a hippocampal electrode was *post hoc* determined based on ripple amplitude and theta phase.^42^

### Epileptiform activity detection

Identification of epileptiform activity was conducted irrespective of the vigilance state based on raw LFP signals obtained from electrodes located centrally in the PFC, PTC and HC using custom-written Python and MATLAB algorithms. To evaluate the frequency of spiking activity, which included spike–wave discharges (SWDs), isolated hippocampal and cortical spikes and giant spikes, the total number of detected events was normalized to the recording duration. Therefore, the frequency of spiking activity was expressed as events per second in hertz. SWDs were quantified from the PFC channels using custom-written algorithms, as described previously.^43,44^ An SWD was defined as an asymmetric complex of at least three cycles of sharp negative–positive going spikes and waves with peak-to-peak amplitudes > 2-fold higher than background LFP, a minimal discharge frequency of 6 Hz, a minimal duration of 1 s and an inter-SWD episode interval of ≥1 s. All candidate SWDs were visually inspected to distinguish real from false-positive SWDs.^32^ To detect isolated spiking activity from PTC and HC channels, first, an automatic spike detection method was employed using the nonlinear energy operator (NEO) combined with automatic NEO thresholding.^45^ After that, giant spikes were identified and extracted from detected cortical and hippocampal spikes. Giant spikes were defined as simultaneous spikes (either positive or negative) present across all channels, with a large amplitude (>±10 SD from the filtered baseline) in at least one hippocampal channel, followed by a positive deflection (‘after hyperpolarization’) lasting >200 ms across all channels.^32^ The remaining spikes were classified into isolated hippocampal or cortical spikes. Isolated hippocampal spikes were detected from HC electrodes as brief (<100 ms) negative-going single spikes that did not have a corresponding spike in the contralateral HC within a ±100 ms window. Similarly, isolated cortical spikes were detected from PTC electrodes using the same criteria.

Additionally, to better characterize the timing of spontaneous isolated spiking activity, we analysed the inter-spike intervals (ISIs), defined as the span of time from one spike to the next in the sequence of spike times recorded from the hippocampal or cortical regions. Due to the relative rarity of events with long ISIs, raw bin counts tend to exhibit greater variability at longer intervals. To address this and provide a more accurate representation of the ISI distribution across time scales, we applied logarithmic binning, which ensures improved resolution and stability in both short and long ISI ranges.

### Vigilance state assessment

For the full vigilance state scoring description, see the method of van Heusden *et al*.^42^ that was used. In brief, behavioural states, including active wake, quiet wake and sleep, were assigned per 5 s epoch based on the delta/theta ratio and the video-tracked velocity of the animal. For fE/I analysis, recordings from quiet wake were used to align with the fE/I analysis approach in humans based on resting-state EEG.^21^ For θ–γ PAC analysis, active wake periods were used based on the identified role of θ–γ PAC during this phase in the context of memory recall^46^ and earlier work in an epilepsy model.^47^

### fE/I analysis

The fE/I algorithm was applied to cleaned LFP data (i.e. processed to exclude movements, technical artefacts and epileptiform spikes) in narrow frequency ranges following the procedure described in Diachenko *et al*.^22^ In brief, LFP signals were bandpass filtered into 16 frequency bands spanning the 1–150 Hz spectrum, 1 band from 1 to 4 Hz and 15 logarithmically spaced bands between 4 and 150 Hz. For each channel within each band, the fE/I ratio was computed from the correlation between the windowed LFP amplitude and its amplitude modulation (i.e. the temporal autocorrelation structure). Analyses were restricted to quiet wake intervals as determined by vigilance state annotations. Intervals from each weekly recording that shared the same annotation were concatenated if they yielded at least 2 min of continuous data, in line with prior recommendations.^22^ Recordings that did not meet this criterion were excluded. Overall, 19% of all weekly recordings were excluded. The fE/I was assigned a missing value (NaN) whenever long-range temporal correlations (LRTCs), assessed via detrended fluctuation analysis (DFA), were absent (i.e. DFA exponent < 0.6). Further details of these algorithms are provided in Supplementary Material S1 and S2 in Diachenko *et al*.^22^

### θ–γ PAC analysis

θ–γ PAC features were assessed using custom-written scripts in MATLAB^48^ based on the modulation index (MI) parameter, as described previously.^49^ Clean LFP signals within active wake intervals from all electrodes located in the PTC and HC were used. First, LFP signals were bandpass filtered for phase in the range of 2–14 Hz with 2 Hz bandwidths and steps of 1 Hz. For the amplitude of cortical channels, LFP signals were bandpass filtered in the range of 40–200 Hz with 2 Hz bandwidths and steps of 1 Hz, and for hippocampal LFP signals in the range of 40–300 Hz with 4 Hz bandwidths and steps of 2 Hz. Next, Hilbert transformation was applied to extract phase and amplitude information. Each phase frequency was binned into 18 bins of 20°, and the average amplitude per each amplitude frequency was calculated inside each bin. A distribution was subsequently obtained by normalizing the average amplitude in each phase bin by the sum over all bins. To compare the resulting phase–amplitude distribution with a uniform distribution, the Kullback–Leibler distance was calculated. The MI was obtained by normalizing this metric to the logarithm of the number of phase bins. For group analyses, cortical and hippocampal θ–γ PAC was expressed as a single value by averaging over θ (4–10 Hz) to low γ (40–90 Hz) and θ to high γ (90–160 Hz), respectively. Hippocampal θ–γ PAC was also expressed as a single value by averaging over θ (4–10 Hz) to total γ (40–300 Hz), as described previously.^47^ MI values were calculated individually for each channel for a total of 10 min for each month (by concatenating weekly recordings of a particular month) in the PTC and HC (i.e. supra-pyramidal, pyramidal and infra-pyramidal). Analyses were performed using custom-written scripts in MATLAB.^48^

### Statistical analysis

#### Bayesian multilevel statistical modelling

Bayesian multilevel models (BMLMs), which provide a probabilistic statistical framework for analysing nested data, were used to model the change in the mean fE/I or θ–γ PAC across the observation window and test for differences between APP/PS1 and WT mice, with comparisons made to the baseline (i.e. 3 months of age). Separate analyses were conducted for each brain region, including the HC (i.e. supra-pyramidal, pyramidal and infra-pyramidal CA1 layers) and PTC. Models were fitted using the *brms* package^50,51^ in R Statistical Software.^52^ Default weakly informative priors provided by *brms* were used, ensuring model flexibility without overfitting. Diagnostic checks were performed to confirm model convergence, reliability and overall fit to the observed data.

After fitting the models, the posterior distributions of the fixed-effect coefficients were examined to assess the impact of each predictor. To evaluate statistical significance, Bayesian *P-*values (hereafter referred to simply as *P*-values or *P*) were computed from the posterior distributions by determining the smaller proportion of posterior samples where the parameter estimate was >0 or <0, then doubling that proportion to obtain a two-tailed *P*-value.

##### fE/I features

For each frequency band of fE/I, a BMLM was fitted with fE/I as the response variable and genotype, age (in months) and their interaction as categorical predictors. To capture baseline variability across individual mice, a mouse-specific intercept was included. This intercept was allowed to vary by channel within each mouse, to account for variability at the electrode level. Finally, a random slope for the effect of age was included at both the mouse and channel levels. Although all 16 frequency bands were systematically analysed (Supplementary Fig. 1), the primary focus in the ‘Results’ section was on narrow-band low-gamma activity in the range of 35–45 Hz because of its biological relevance in Alzheimer's disease.^53-55^ To correct for comparisons across the 16 bands, we pre-specified a per-comparison significance level *α* < 0.01 to minimize false positives. We used an *ad hoc* correction approach over formal *post hoc* multiple comparison corrections for its simplicity and interpretability. Results of which 0.01 ≤ *P* < 0.05 were reported as marginally significant, reflecting trends that may be meaningful. Note that while these marginal findings were identified in the statistical analyses, not all of them were necessarily discussed in the ‘Results’ section.

##### θ–γ PAC features

For θ–γ PAC (separately for θ–low-γ and θ–high-γ PAC), a BMLM was fitted with genotype, age (in months) and their interaction as categorical predictors. A random intercept was included for each mouse, and this intercept was allowed to vary by channel within each mouse. A random slope for age was not included because θ–γ PAC values were calculated on a monthly basis (i.e. by concatenating weekly recordings within a particular month). For consistency with the fE/I analysis, we used *α* < 0.01 while reporting marginally significant results where 0.01 ≤ *P* < 0.05.

#### Correlation analyses

All correlation analyses were conducted separately for each brain region, including the three hippocampal CA1 layers and PTC.

##### Correlation between spectral fE/I and θ–γ PAC data

To match θ–γ PAC values (calculated per channel per month) with fE/I values (calculated per channel per week), the fE/I ratio for each frequency interval was first averaged at the electrode level across the 4 weeks of a given month. To address for non-independence in the data, repeated measures correlation,^56^ implemented in the *rmcorr* function of the *Pingouin*^57^ Python package, was used to estimate the common within-mouse association between fE/I and θ–γ PAC, while adjusting for inter-mouse variability. Repeated measures for each mouse consisted of monthly paired electrode-level values of fE/I and θ–γ PAC over the 3- to 11-month observation period. The correlation coefficient, bounded between −1 and 1, was calculated separately for WT and APP/PS1 mice and for each frequency band of fE/I, reflecting the strength and direction of the linear association between fE/I and θ–γ PAC. The primary significance level was similarly set to *α* < 0.01.

##### Correlation between spectral fE/I and epileptiform features

Correlations were performed separately for each spiking event type only in APP/PS1 mice, as WT mice did not have enough data to support correlation analyses. To match epileptiform spiking frequency values (computed across all channels per week) with fE/I (computed per channel per week), fE/I ratios for each frequency interval were grouped by mouse and week, then averaged across channels to yield a single fE/I ratio per mouse per week. Repeated measures correlation^56,57^ was used to determine the within-mouse association between spiking activity and fE/I. Repeated measures for each mouse consisted of weekly paired mouse-level values of fE/I and the frequency of spiking activity over the 3- to 11-month observation period. Only weeks with non-zero spiking frequency were included. The primary significance level was again set to *α* < 0.01.

##### Correlation between θ–γ PAC and epileptiform features

Correlations between θ–γ PAC and epileptiform features were performed separately for each spiking event type only in APP/PS1 mice, as WT mice did not have enough epileptiform data to support correlation analyses. To match epileptiform spiking frequency values (computed across all channels per week) with θ–γ PAC (computed per channel per month), θ–γ PAC values and spike frequencies were grouped by mouse and month, then averaged to yield a single pair of values per mouse per month. Repeated measures correlation^56,57^ was used to determine the within-mouse association between spiking activity and θ–γ PAC. Repeated measures for each mouse consisted of monthly paired mouse-level values of θ–γ PAC and spiking frequency over the 3- to 11-month observation period. Only months with non-zero spiking frequency were included. The primary significance level was again set to *α* < 0.01.

## Results

To explore the potential of fE/I as a biomarker of network E/I imbalance in early stages of Alzheimer's disease, we computed fE/I from LFP signals recorded during quiet wakefulness in the HC and PTC (Fig. 1A, i) of freely behaving APP/PS1 mice between 3 and up to and including 11 months of age (hereafter referred to as ‘between 3 and 11 months of age’; Fig. 1A, ii) and compared the results to those from WT littermates. This time range was chosen since at 4 months, APP/PS1 mice have no detectable amyloid plaques yet, while impairments in inhibitory transmission and cognition start to occur around that age.^31^ fE/I features were correlated to other measures of E/I network excitability, i.e. θ–γ PAC, assessed during active waking, and epileptiform activity, assessed across all vigilance states.

### fE/I as a biomarker of hippocampal E/I imbalance in APP/PS1 mice

To demonstrate that the fE/I algorithm can infer E/I ratios from mouse LFP, we showcased the analysis for signals recorded in the same HC region in one APP/PS1 mouse and one WT mouse at both 3 and 6 months of age (Fig. 1A, ii, arrows). First, narrow-band low-gamma activity, which has been reported to be altered in Alzheimer's disease models and patients,^55^ was extracted in the range of 35–45 Hz, after which the fE/I algorithm was applied as described earlier^22^ (Fig. 1B). Visually, no differences in the amplitude modulation of LFP low-gamma oscillations were observed between the two mice at 3 months (Fig. 1C, i). At 6 months, visibly larger amplitude fluctuations were detected in the signal from the APP/PS1 mouse compared to that from the WT mouse and compared to the signal from the same APP/PS1 mouse at 3 months (Fig. 1C, ii and Fig. 1C, ii versus i, respectively). Next, LRTCs of these modulations were quantified using the DFA exponent (*β*) of the fluctuation function, whereby a *β* value of >0.6 indicates the presence of LRTCs,^21,22^ which was observed for both mice at 3 and 6 months (Fig. 1D, i and ii, insets). Next, fE/I values were derived. At 3 months, both mice were found to exhibit near-balanced E/I ratios as indicated by around zero correlations between the amplitude and the fluctuation function, yielding fE/I values being close to 1 (Fig. 1D, i). At 6 months, however, a strong negative correlation between the amplitude and the fluctuation function was observed for the APP/PS1 but not the WT mouse, which resulted in a strongly increased fE/I value > 1 for the APP/PS1 animal, indicating a specifically increased E/I ratio for the HC in this APP/PS1 mouse at this age (Fig. 1D, ii).

Next, hippocampal fE/I values were computed for all mice and HC electrodes and compared between genotypes at monthly intervals between 3 and 11 months of age (i.e. based on the recordings starting at 3 months and ending just before 12 months of age), which revealed higher fE/I values in APP/PS1 mice at 6 months compared to values of WT mice at these age windows (Table 1). Notably, an increased variability of the fE/I values in APP/PS1 mice was observed at 4 months (Table 1). Overall, these findings support the potential of the fE/I algorithm to infer network E/I ratios from LFP signals in mice and highlight its capability of detecting genotype differences in the mouse brain excitatory–inhibitory network.

**Table 1:** Differences in low-gamma fE/I between APP/PS1 and WT mice in the pyramidal layer of hippocampal CA1

Age (months)	Genotype	*N*_mice_^a^	*N*_el_^b^	${\bar{N}}_{\mathrm{el}/\mathrm{mouse}}$ ^c^	$\bar{\mathrm{fE}/I}$ ^d^	$\Delta \bar{\mathrm{fE}/I}$ ^e^	ΔVar(fE/I)^f^
3	WT APP/PS1	10 (10) 8 (6)	68 (57%) 27 (49%)	6.8 (58%) 4.5 (58%)	0.996 0.988	−0.008	0.013
4	WT APP/PS1	9 (8) 9 (8)	74 (65%) 50 (63%)	9.2 (64%) 6.2 (61%)	0.989 0.934	−0.055	0.036*
5	WT APP/PS1	10 (10) 9 (9)	102 (84%) 59 (65%)	10.2 (77%) 6.6 (64%)	0.989 1.035	0.045	0.005
6	WT APP/PS1	9 (9) 9 (9)	86 (75%) 75 (77%)	9.6 (71%) 8.3 (74%)	0.938 1.143	0.205**	0.007
7	WT APP/PS1	10 (10) 9 (9)	85 (75%) 77 (83%)	8.5 (71%) 8.6 (79%)	1.013 1.055	0.043	−0.009
8	WT APP/PS1	10 (10) 8 (8)	106 (82%) 66 (76%)	10.6 (77%) 8.2 (82%)	1.024 1.041	0.017	−0.016
9	WT APP/PS1	10 (10) 8 (8)	102 (80%) 67 (81%)	10.2 (80%) 8.4 (77%)	0.974 1.012	0.038	0.039
10	WT APP/PS1	10 (10) 8 (8)	110 (83%) 72 (88%)	11 (85%) 9.0 (92%)	1.009 1.056	0.046	0.001
11	WT APP/PS1	10 (10) 8 (8)	89 (78%) 83 (93%)	8.9 (82%) 10.4 (93%)	1.024 1.053	0.029	−0.007

^*^ *P* < 0.05. **P < 0.01.

^a^The total number of mice. In parentheses, the total number of mice with the computed fE/I ratio.

^b^The total number of electrodes available for statistical analyses per month, excluding electrodes with missing fE/I ratio. In parentheses, the total percentage of electrodes with the computed fE/I ratio.

^c^The mean number of electrodes with the computed fE/I ratio per mouse. In parentheses, the percentage of electrodes with computed fE/I per mouse.

^d^The mean fE/I ratio. Monthly averages were calculated across mice by first averaging all computed fE/I ratios from the four weekly measurements of a particular month per electrode and then across electrodes per mouse.

^e^The difference in the mean fE/I ratio between genotypes. Significance (false discovery rate-corrected across nine time points, *q* = 0.05) was determined using either the unpaired Student’s *t*-test (equal variance) or the Welch’s *t*-test (unequal variance).

^f^The difference in the variance of fE/I ratio between genotypes. Significance was assessed using Levene’s test for equality of variances.

### fE/I indicates a shift towards excitation-dominated low-gamma activity in the hippocampal CA1 region of APP/PS1 mice

**Figure 2:**
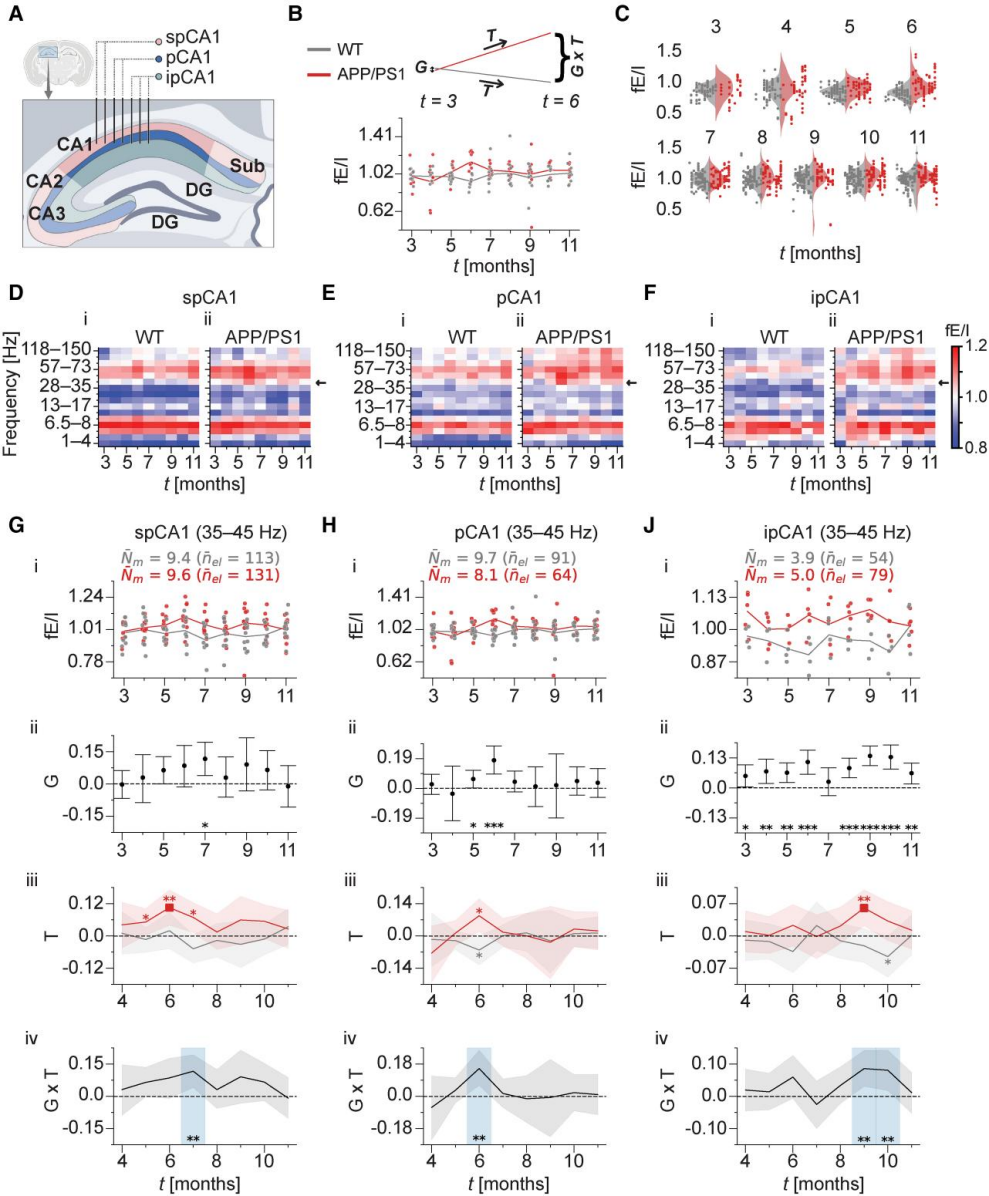
**Elevated fE/I indicates excitation-dominated activity of low-gamma oscillations in the hippocampal CA1 region of APP/PS1 mice.** (**A**) Schematic representation of electrode placement across the hippocampal CA1 layers (spCA1, pCA1 and ipCA1). (**B**) Age-related low-gamma fE/I (35–45 Hz) in pCA1. Data points represent individual mice and are shown for each genotype at each month, with lines representing the corresponding genotype means. Each data point at each month reflects a mouse’s fE/I value, first averaged across weeks at the mouse–electrode level, then averaged across electrodes at the mouse level. The inset explains the effects of genotype (G), age (T) and their interaction (G × T) for an example time point, estimated using BMLM statistical modelling. (**C**) Violin plots of low-gamma fE/I by month and genotype to illustrate the raw biomarker values and different sources of variation analysed with BMLM. Vertically aligned dots in each violin represent electrodes from the same mouse. (**D–F**) Age-related, genotype-averaged fE/I across the 1–150 Hz spectrum (*y*-axis) for LFP in the HC. Data are shown from 3 to 11 months of age (*x*-axis). fE/I values were first averaged at the mouse–electrode level across weeks for a given month, then at the mouse level across electrodes per month. Panels show data for spCA1 (**D**), pCA1 (**E**) and ipCA1 (**F**). Plots (i) and (ii) within each panel represent the WT and APP/PS1 genotypes, respectively. (**G–I**) Elevated low-gamma fE/I reflects excitation-dominated activity in the HC of APP/PS1 mice. (i) Age-related fE/I for low-gamma (35–45 Hz) oscillations in spCA1 (**G**), pCA1 (**H**) and ipCA1 (**I**). Data points represent individual mice (similar aggregation as in **B**). ${\bar{n}}_{\mathrm{el}}$ and ${\bar{N}}_{m}$ denote the average number of total electrodes and mice with computed fE/I per genotype across months, respectively. BMLM was used to estimate the genotype effect (ii), the age effect (iii) and their interaction (iv). Dots in (ii) and lines in (iii, iv) represent posterior mean parameter estimates. In total, 2208 data points were analysed in spCA1, 1398 in pCA1 and 1276 in ipCA1. Error bars in (ii), shaded areas in (iii), and the dark shaded area in (iv) indicate 95% CIs. Time points with significant differences are indicated by asterisks in (ii), filled squares and asterisks coloured by genotype in (iii) and shaded regions and asterisks in (iv). *P  *<* 0.05; **P  *<* 0.01; ***P  *<* 0.001. WT, wild-type; APP/PS1, APPswe/PSEN1de9; DG, dentate gyrus; Sub, subiculum; CA1, CA2 and CA3, dorsal hippocampal subfields that form part of the Cornu Ammonis (CA) region; spCA1, supra-pyramidal layer of CA1; pCA1, pyramidal layer of CA1; ipCA1, infra-pyramidal layer of CA1; HC, hippocampus; BMLM, Bayesian multilevel modelling; LFP, local field potential; fE/I, functional excitation–inhibition. The depicted coronal brain view with zoomed-in hippocampal layers and electrode configuration in **A** was created with BioRender.com (Krivoshein, G. (2025); https://BioRender.com/464v286).

To further investigate age-related changes in network E/I ratios in the HC, fE/I was analysed separately for LFP signals obtained from the supra-pyramidal, pyramidal and infra-pyramidal CA1 layers (Fig. 2A). fE/I values were extracted from narrow-band signals spanning the 1–150 Hz frequency spectrum, using 1 bin between 1 and 4 Hz and 15 log-spaced bins between 4 and 150 Hz, as described previously.^22^ The age-related, genotype-averaged fE/I values in the three CA1 layers of WT and APP/PS1 mice are shown in Fig. 2D–F, revealing distinct fE/I values across the frequency spectrum. Separately for each frequency band of fE/I, multilevel statistical modelling was used to estimate the effects of interest, while accounting for the nested data structure and group-level effects associated with both individual mice and electrodes. Figure 2B and C provides an explanation of these estimates. The effects were estimated of (i) genotype (i.e. APP/PS1 and WT mice at each month), (ii) age (i.e. within each genotype relative to 3 months) and (iii) their interaction (i.e. genotype differences in age-related fE/I changes relative to 3 months). For each frequency band, the effects are summarized by their posterior mean parameter estimates in Supplementary Fig. 1. Their 95% confidence intervals (95% CI) and *P*-values, as well as the total number of analysed data points per frequency band, can be found in Supplementary Tables 1–3 for the supra-pyramidal, pyramidal and infra-pyramidal CA1 layers, respectively.

Focusing on the 35–45 Hz range, for LFP recorded from the supra-pyramidal and pyramidal CA1 layers, the fE/I values were close to 1 at 3 months in both genotypes (Fig. 2G and H, i and ii), indicating balanced E/I at baseline. Notably, in the supra-pyramidal layer, WT mice showed no age-related fE/I changes throughout the observation period (Fig. 2G, iii). However, in that CA1 layer, APP/PS1 mice exhibited a transient elevation in fE/I above 1 between 5 and 7 months relative to baseline [Fig. 2G, iii; *P*  *<* 0.01 at 6 months, with trends at 5 (*P*  *=* 0.046) and 7 months (*P*  *=* 0.020)]. The age-related fE/I elevation in supra-pyramidal CA1 in APP/PS1 animals was pronounced at 7 months compared to the corresponding fE/I change in WT mice (Fig. 2G, iv; *P*  *<* 0.01), with higher fE/I values in APP/PS1 than in WT mice (Fig. 2G, ii; a borderline difference with *P*  *=* 0.010). In the pyramidal CA1 layer, similar effects emerged at 6 months. At this age, APP/PS1 mice showed an uptrend in fE/I above 1 relative to baseline (Fig. 2H, iii; *P*  *=* 0.015), whereas WT mice tended to exhibit a decrease in fE/I below 1 (Fig. 2H, iii; *P*  *=* 0.045). Compared to the fE/I downtrend in the pyramidal CA1 layer in WT mice, the fE/I uptrend in this layer in APP/PS1 mice was pronounced during this age period (Fig. 2H, iv; *P*  *<* 0.01), with APP/PS1 mice displaying higher fE/I than WT mice [Fig. 2H, ii; *P*  *<* 0.001, with a trend at 5 months (*P*  *=* 0.041)]. Although the effect in fE/I in this layer at 6 months in APP/PS1 mice [95% CI = (0.0198, 0.1648)] was stronger than that in WT mice [95% CI = (−0.1274, −0.0009)], it is possible that the latter contributed to the observed genotype difference in the age-related fE/I changes in Fig. 2H, iv. Nevertheless, because the fE/I reduction in WT mice was near the 0.05 cut-off, with the upper 95% CI bound almost touching zero, and did not replicate in any of the adjacent layers (Fig. 2G and I), we interpreted it as experimental variability or noise rather than a true age-related change.

Finally, for the infra-pyramidal CA1 layer, fE/I values in the 35–45 Hz range were marginally higher in APP/PS1 compared to WT mice at the 3-month baseline (Fig. 2I, ii; *P*  *=* 0.036). In APP/PS1 mice, fE/I remained above 1 between 4 and 8 months relative to baseline (Fig. 2I, iii), exhibited a further transient increase at 9 months (Fig. 2I, iii; *P*  *<* 0.01) and returned to baseline by 11 months. In contrast, WT mice had fE/I values below 1 at baseline for the infra-pyramidal CA1 layer, which did not change with age (Fig. 2I, iii), except for a marginal decrease at 10 months (Fig. 2I, iii; *P*  *=* 0.047). Genotype differences in age-related fE/I changes were pronounced at 9 and 10 months (Fig. 2I, iv; both comparisons with *P*  *<* 0.01), consistent with the transient fE/I alterations seen in both groups. Overall, low-gamma fE/I was higher for the infra-pyramidal CA1 layer in APP/PS1 than in WT mice throughout the entire observation period, except at 7 months (Fig. 2I, ii).

Notably, in the infra-pyramidal CA1 layer (but not in the supra-pyramidal and pyramidal layers), early genotype differences were also observed for fE/I in the delta– (1–4 Hz) and beta–low-gamma (28–35 Hz) frequency ranges (Supplementary Fig. 1G and H). Overall, results in the beta–low-gamma range indicated increased yet more balanced E/I (i.e. fE/I close to 1) in APP/PS1 mice compared to inhibition-dominated E/I (i.e. fE/I < 1) in WT mice during the entire observation period (Supplementary Fig. 1H, ii; all but one comparison with *P*  *<* 0.001). In the delta band, APP/PS1 mice exhibited a reduction in inhibition (i.e. fE/I shift to ≥1) between 4 and 8 months compared to baseline (Supplementary Fig. 1G, iii; all but one comparison with *P*  *<* 0.001). At all these time points except at 5 months, differences in fE/I were observed between genotypes, revealing increased fE/I values for APP/PS1 mice (Supplementary Fig. 1G, ii; all but one comparison with *P*  *<* 0.001). The age-related fE/I increases observed in the infra-pyramidal CA1 layer in APP/PS1 mice contrasted with the unaltered fE/I in WT mice during these age windows (Supplementary Fig. 1G, iv; all but one comparison with *P*  *<* 0.001). These findings suggest that early impairments in E/I balance may particularly emerge in the infra-pyramidal CA1 layer, where reduced network inhibition is observed across multiple frequency bands.

Overall, the involvement of 35–45 Hz oscillations in hippocampal E/I imbalances in APP/PS1 mice appears consistent in all three investigated CA1 layers. Taken together, our findings reveal a shift towards excitation-dominated (fE/I > 1) narrow-band low-gamma activity in the supra-pyramidal and pyramidal CA1 layers of APP/PS1 mice specifically at 6–7 months, with an elevated network E/I ratio detectable in the infra-pyramidal CA1 layer as early as 3–4 months.

### Age-related impairments of θ–low-γ PAC in the hippocampal CA1 region of APP/PS1 mice

**Figure 3:**
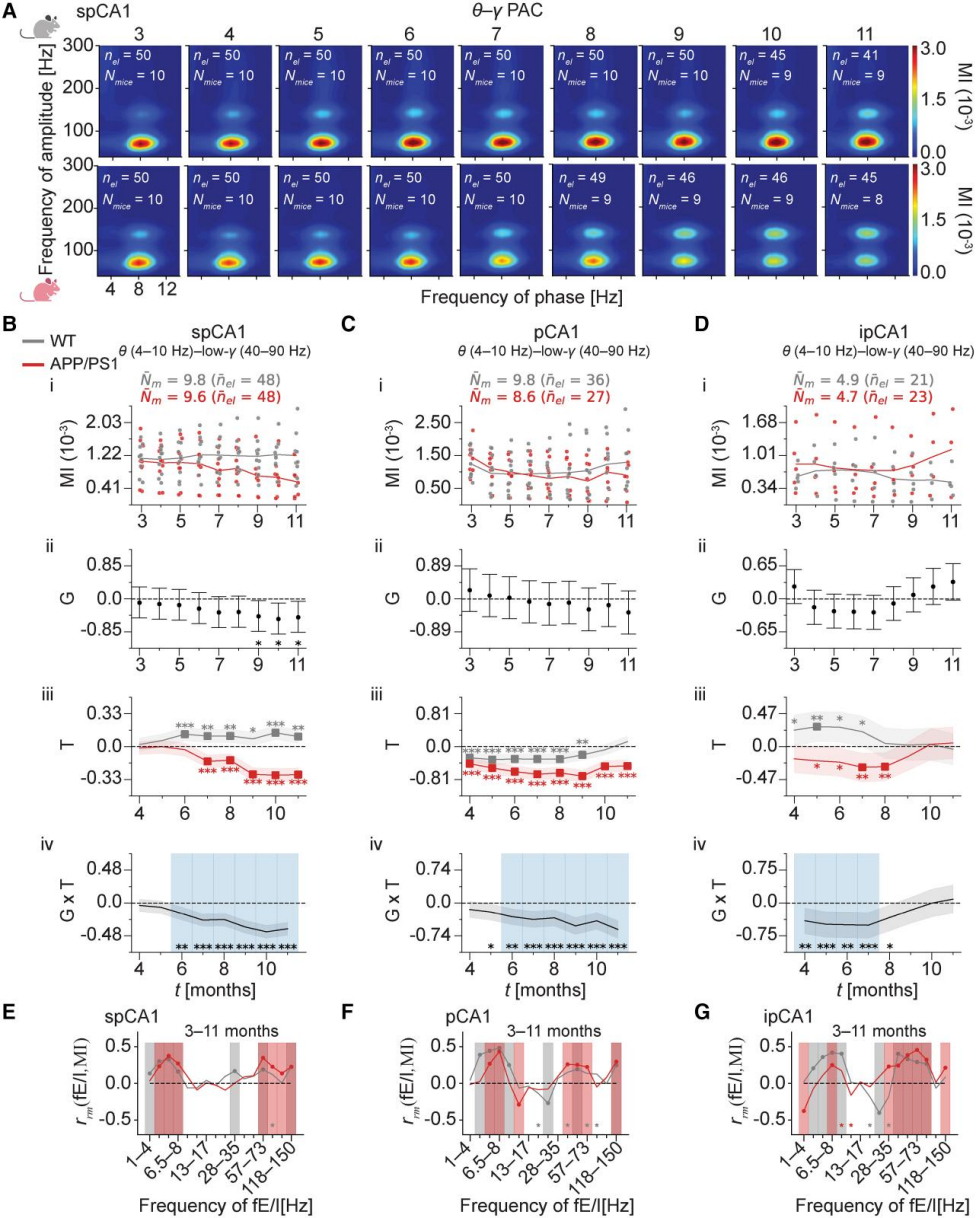
**Age-related impairment of θ–low-γ PAC in the hippocampal CA1 region of APP/PS1 mice.** (**A**) Average θ–γ PAC comodulograms of hippocampal (spCA1) LFP in WT (top row) and APP/PS1 mice (bottom row) during active wakefulness from 3 to 11 months of age (columns). See Supplementary Fig. 2A and B for pCA1 and ipCA1, respectively. All subplots share the same *x*-axis and *y*-axis. (**B–D**) Age-related impairment of θ–low-γ PAC for spCA1 (**B**), pCA1 (**C**) and ipCA1 (**D**). (i) Age-related θ–low-γ PAC. Data points represent individual mice and are shown for each genotype at each month, with lines representing the corresponding genotype means. Each data point at each month reflects a mouse’s θ–low-γ PAC value, averaged across electrodes at the mouse level per month. ${\bar{n}}_{\mathrm{el}}$ and ${\bar{N}}_{m}$ denote the average number of total electrodes and mice with computed θ–γ PAC per genotype across months, respectively. BMLM was used to estimate the genotype effect (ii), the age effect (iii) and their interaction (iv). Dots in (ii) and lines in (iii, iv) represent posterior mean parameter estimates. In total, 874 data points were analysed in spCA1, 572 in pCA1 and 401 in ipCA1. Error bars in (ii), shaded areas in (iii), and the dark shaded area in (iv) indicate 95% CIs. Time points with significant differences are indicated by asterisks in (ii), filled squares and asterisks coloured by genotype in (iii) and shaded regions and asterisks in (iv). (**E–G**) A repeated measures correlation test was used to estimate the correlation coefficient, *r*_rm_(fE/I, MI) (*y*-axis), between monthly paired fE/I and θ–low-γ PAC, which is plotted across the fE/I frequency spectrum (*x*-axis) for WT and APP/PS1 mice, for spCA1 (**E**), pCA1 (**F**) and ipCA1 (**G**). Filled circles and shaded vertical regions indicate significant (*P*  *<* 0.01) correlations. One asterisk marks marginal significance (0.01 ≤ *P* < 0.05). *P  *<* 0.05; **P  *<* 0.01; ***P  *<* 0.001. WT, wild-type; APP/PS1, APPswe/PSEN1de9; θ–γ PAC, theta–gamma phase–amplitude coupling; spCA1, supra-pyramidal layer of CA1; pCA1, pyramidal layer of CA1; ipCA1, infra-pyramidal layer of CA1; BMLM, Bayesian multilevel modelling; G, genotype effect; T, age effect; G × T, interaction effect between genotype and age; fE/I, functional excitation–inhibition; LFP, local field potential; MI, modulation index (see ‘θ–γ PAC analysis’ section in the ‘Materials and methods’ section).

To provide better insight in the underlying E/I imbalances contributing to the observed fE/I changes in APP/PS1 mice, the analysis was expanded to θ–γ PAC, a metric strongly relying on hippocampal–cortical inhibitory network activity^34^ that is impaired in Alzheimer's disease patients^38^ and mouse models.^39,41^ Hence, we computed θ–γ PAC from the LFP signals recorded from the HC of APP/PS1 mice over the period between 3 and 11 months of age and compared the results to those of WT mice of the same age. Since patterns of θ–γ PAC differ across the hippocampal layers,^58^ θ–γ PAC was again separately analysed for the supra-pyramidal, pyramidal and infra-pyramidal CA1 layers. Similar to the fE/I algorithm, θ–γ PAC analyses took into account effects of genotype, age and their interaction, using multilevel statistical modelling. As during quiet wakefulness prominent θ oscillations are absent and associated with no or weak modulation of γ activity,^59^ the analysis was restricted to the active awake state (Fig. 3A for the supra-pyramidal CA1 layer; see Supplementary Fig. 2 for the average θ–γ PAC comodulograms of LFP in the pyramidal and infra-pyramidal layers). For all hippocampal signals, θ–low-γ (40–90 Hz) PAC was computed. For signals recorded from the supra-pyramidal CA1 layer, θ–high-γ (90–160 Hz) activity was also computed (Fig. 3A). In contrast, within the pyramidal and infra-pyramidal CA1 layers, modulation in the θ–high-γ frequency range was not consistently observed, unlike θ–low-γ, and instead the coupling from θ to γ extended across higher γ oscillations (Supplementary Fig. 2A and B). Therefore, θ–total-γ (40–300 Hz) PAC was also computed for these layers.

Over the 3- to 11-month observation window, no genotype differences in θ–low-γ PAC were observed in the pyramidal and infra-pyramidal CA1 layers (Fig. 3C and D, ii). Marginal differences, however, were observed between 9 and 11 months for the supra-pyramidal CA1 layer, with APP/PS1 mice showing lower θ–low-γ PAC than WT mice (Fig. 3B, ii; all comparisons with 0.01 ≤ *P* < 0.05). Further, distinct age-related changes emerged at earlier time points that differed for APP/PS1 and WT mice and varied across the CA1 layers.

For the supra-pyramidal CA1 layer, WT mice exhibited a steady increase in θ–low-γ PAC over the observation window, with progression between 6 and 11 months relative to the 3-month baseline (Fig. 3B, iii; all comparisons with *P*  *<* 0.01 or *P*  *<* 0.001, except at 9 months where 0.01 ≤ *P* < 0.05). APP/PS1 mice also maintained baseline-level θ–low-γ PAC levels between 4 and 6 months but showed a marked decline between 7 and 11 months (Fig. 3B, iv; all comparisons with *P*  *<* 0.001). Overall, for supra-pyramidal CA1, genotype differences in the age-related θ–low-γ PAC changes were observed between 6 and 11 months (Fig. 3B, iv; all but one comparison with *P*  *<* 0.001). For LFP recorded from the pyramidal CA1 layer, both genotypes experienced a decrease in θ–low-γ PAC levels beginning at 4 months (Fig. 3C, iii; all but one comparison with *P*  *<* 0.001). In WT mice, however, θ–low-γ PAC returned to baseline by 10 months. Compared to the age-related θ–low-γ PAC decreases in WT mice, APP/PS1 mice displayed a more pronounced reduction in θ–low-γ PAC between 6 and 11 months (Fig. 3C, iv; all but one comparison with *P*  *<* 0.001). Finally, for LFP recorded from the infra-pyramidal CA1 layer, APP/PS1 mice showed a decrease in θ–low-γ PAC relative to baseline, most notably between 7 and 8 months (Fig. 3D, iii; *P*  *<* 0.01), followed by a subsequent return to the baseline level. In WT mice, θ–low-γ PAC gradually increased between 4 and 7 months, with the most prominent rise at 5 months (Fig. 3D, iii; *P*  *<* 0.01), before returning to baseline from 8 months onwards. Genotype differences in the age-related θ–low-γ PAC changes were observed between 4 and 7 months (Fig. 3D, iv; all comparisons with *P*  *<* 0.01 or *P<* 0.001).

For the θ–total-γ PAC in the pyramidal and infra-pyramidal CA1 layers, the results were generally similar to yet less prominent than those observed for the θ–low-γ PAC (Supplementary Fig. 2C and D). As for the θ–high-γ PAC in the supra-pyramidal CA1 layer, no genotype differences were observed across the observation period (Supplementary Fig. 3, ii). Differences in age-related θ–high-γ PAC changes between APP/PS1 and WT mice were pronounced between 10 and 11 months (Supplementary Fig. 3, iv; both comparisons with *P*  *<* 0.001). Taken together, these results indicate that alterations in θ–γ PAC within the hippocampal CA1 region are both age- and layer-specific.

### CA1 layer-related θ and γ fE/I correlates with θ–γ PAC also during periods of age-related θ–γ PAC impairments

To compare θ–γ PAC and fE/I at different frequencies as metrics of network E/I balance, we correlated the two separately for APP/PS1 and WT mice. Because the data included repeated measures for each mouse [i.e. monthly paired electrode-level values of fE/I and θ–γ (40–90 Hz) PAC], repeated measures correlation^56^ was used to assess the common within-mouse linear association. Over the 3- to 11-month observation period, we found that fE/I in the theta and gamma bands positively correlated with θ–γ PAC in all three CA1 layers for both genotypes (Fig. 3E–G). Although the specific boundaries of the theta and gamma bands varied by layer, similar significant correlations emerged when using the average fE/I values for the theta (4–5 Hz, 5–6.5 Hz, 6.5–8 Hz and 8–10.5 Hz) and gamma ranges (35–45 Hz, 45–57 Hz, 57–73 Hz and 73–92.5 Hz) (data not shown). Moreover, these correlations were observed during the age windows when APP/PS1 mice exhibited age-related impairments in θ–γ PAC compared to WT mice, specifically between 6 and 11 months for the supra-pyramidal and pyramidal layers and between 4 and 8 months for the infra-pyramidal layer (Supplementary Figs 4A–C, i–iv; all but three comparisons with *P*  *<* 0.001). Overall, these results support a positive association between hippocampal E/I balance in neuronal circuits generating theta and gamma oscillations and the strength of θ–γ PAC, and importantly, this relationship persists even during periods of age-related impairments in θ–γ PAC in APP/PS1 mice.

### Cortical θ–low-γ PAC is attenuated in 3- to 11-month-old APP/PS1 mice and correlates with θ and low-γ fE/I

**Figure 4:**
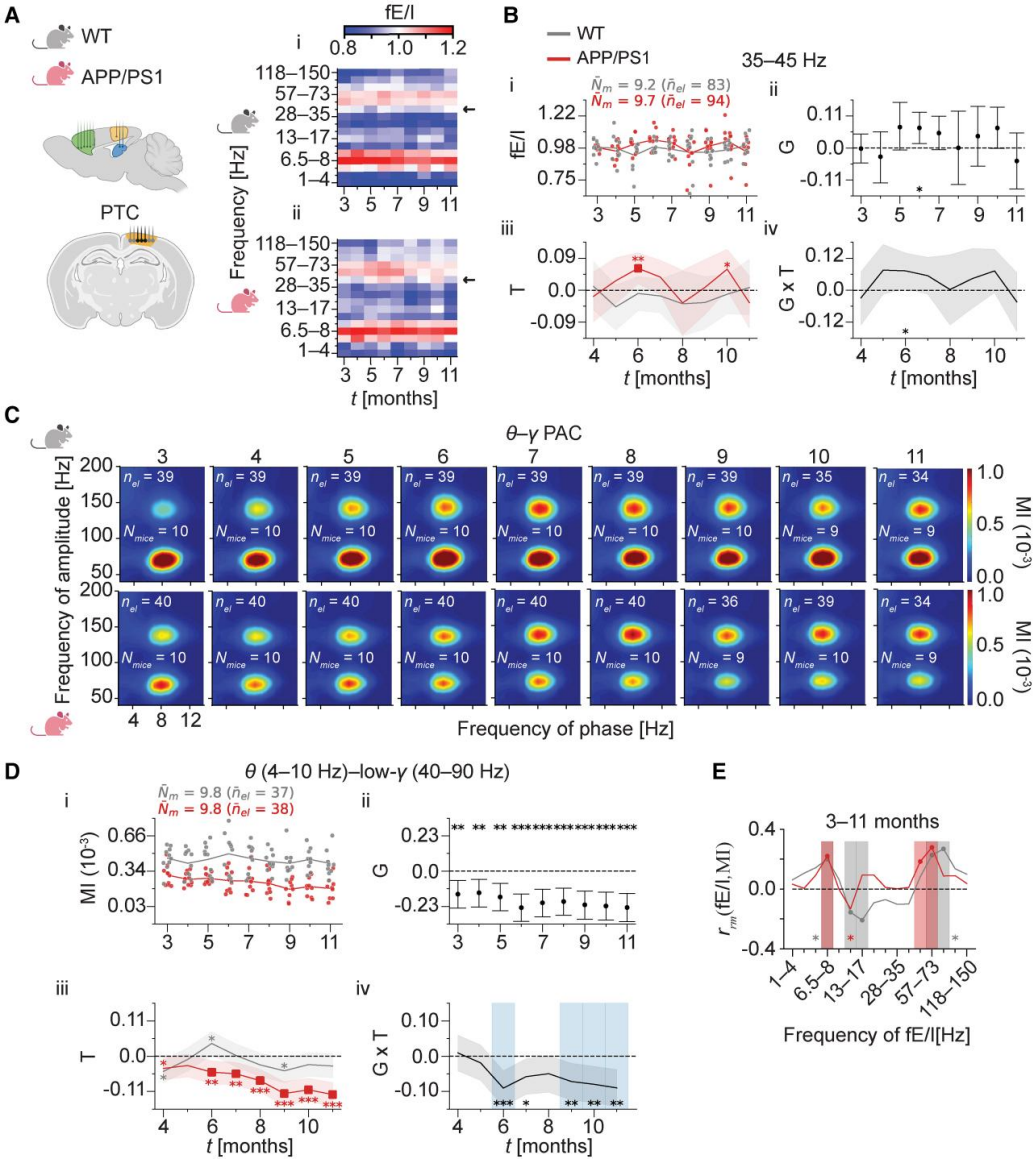
**Impairment of θ–low-γ PAC in the PTC of 3- to 11-month-old APP/PS1 mice.** (**A**) Age-related, genotype-averaged fE/I across the 1–150 Hz spectrum (*y*-axis) for LFP in the PTC. Data are shown from 3 to 11 months of age (*x*-axis). fE/I values were first averaged at the mouse–electrode level across weeks for a given month and then at the mouse level across electrodes per month. Plots (i) and (ii) represent the WT and APP/PS1 genotypes, respectively. (**B**) Elevated low-gamma fE/I in the PTC of 6-month-old APP/PS1 mice. (i) fE/I for low-gamma (35–45 Hz) oscillations over 3–11 months. Data points represent individual mice and are shown for each genotype at each month, with lines representing the corresponding genotype means. Each data point at each month reflects a mouse’s fE/I value, first averaged across weeks at the mouse–electrode level, then averaged across electrodes at the mouse level. ${\bar{n}}_{\mathrm{el}}$ and ${\bar{N}}_{m}$ denote the average number of total electrodes and mice with computed fE/I per genotype across months, respectively. BMLM was used to estimate the genotype effect (ii), the age effect (iii) and their interaction (iv). Dots in (ii) and lines in (iii, iv) represent posterior mean parameter estimates. In total, 1606 data points were analysed. Error bars in (ii), shaded areas in (iii), and the dark shaded area in (iv) indicate 95% CIs. Time points with significant differences are indicated by asterisks in (ii), filled squares and asterisks coloured by genotype in (iii) and shaded regions and asterisks in (iv). (**C**) Average θ–γ PAC comodulograms of cortical LFP in WT (top row) and APP/PS1 mice (bottom row) during active wakefulness from 3 to 11 months of age (columns). All subplots share the same *x*-axis and *y*-axis. (**D**) (i) Each data point at each month reflects a mouse’s θ–low-γ PAC value, averaged across electrodes at the mouse level per month. ${\bar{n}}_{\mathrm{el}}$ and ${\bar{N}}_{m}$ denote the average number of total electrodes and mice with computed θ–low-γ PAC per genotype across months, respectively. BMLM was used to estimate the genotype effect (ii), the age effect (iii) and their interaction (iv). Dots in (ii) and lines in (iii) and (iv) represent posterior mean parameter estimates. In total, 691 data points were analysed. (**E**) A repeated measures correlation test was used to estimate the correlation coefficient, *r*_rm_(fE/I, MI) (*y*-axis), between monthly paired fE/I and θ–low-γ PAC across the fE/I frequency spectrum (*x*-axis) for WT and APP/PS1 mice. Filled circles and shaded vertical regions indicate significant (*P*  *<* 0.01) correlations. One asterisk marks marginal significance (0.01 ≤ *P* < 0.05). *P  *<* 0.05; **P  *<* 0.01; ***P  *<* 0.001. WT, wild-type; APP/PS1, APPswe/PSEN1de9; θ–γ PAC, theta–gamma phase–amplitude coupling; PTC, parietal cortex; BMLM, Bayesian multilevel modelling; G, genotype effect; T, age effect; G × T, interaction effect between genotype and age; fE/I, functional excitation–inhibition; LFP, local field potential; MI, modulation index (see ‘θ–γ PAC analysis’ section in the ‘Materials and methods’ section). The depicted coronal and sagittal brain views with electrode configuration in **A** were created with BioRender.com (Krivoshein, G. (2025); https://BioRender.com/pgyulnz).

We further applied the algorithms of fE/I and θ–γ PAC to cortical LFP signals obtained from PTC and estimated the effects of genotype, age and their interaction as before. At baseline, a balanced fE/I around 1 was observed for the low-gamma (35–45 Hz) band in both genotypes (Fig. 4B, i and ii). While no fE/I changes were observed in WT mice relative to baseline at any of the time points (Fig. 4B, iii), APP/PS1 mice exhibited a transient increase in fE/I > 1 for the low-gamma band at 6 months (Fig. 4B, iii; *P*  *<* 0.01). At this age, a marginal genotype difference was observed in the age-related fE/I changes (Fig. 4B, ii; *P*  *=* 0.034), with fE/I values trending higher in APP/PS1 than in WT mice (Fig. 4B, ii; *P*  *=* 0.016). While these effects did not meet our primary significance threshold of 0.01, they are consistent with the findings in the pyramidal CA1 layer during the same age window, albeit less pronounced. For all effects, their 95% CI and *P*-values, as well as the total number of analysed data points per frequency band, can be found in Supplementary Table 4.

Profound θ–γ PAC [with coupling to both low (40–90 Hz) and high (90–160 Hz) gamma] was evident in the PTC (Fig. 4C). In the PTC, θ–low-γ PAC in particular is likely to reflect inhibitory network interactions predominantly occurring in the HC, as previously demonstrated in a transgenic epilepsy mouse model with impaired GABAergic inhibition.^47^ Consistent with this, APP/PS1 mice showed reduced θ–low-γ PAC compared to WT mice across the entire observation window (Fig. 4D, ii; all comparisons with *P*  *<* 0.01 or *P*  *<* 0.001), whereas no genotype differences were observed in θ–high-γ PAC (data not shown). In both genotypes, a transient, marginal decrease in θ–low-γ PAC relative to baseline was observed at 4 months (Fig. 4D, iii; both comparisons with 0.01 ≤ *P* < 0.05). From 6 months onwards, however, APP/PS1 mice exhibited pronounced reductions in θ–low-γ PAC compared to the baseline level (Fig. 4D, iii; all comparisons with *P*  *<* 0.01 or *P*  *<* 0.001). In WT mice, θ–low-γ PAC remained unchanged (Fig. 4D, iii), except for an additional transient, marginal increase at 6 months (Fig. 4D, iii; *P*  *=* 0.032) and a decrease at 9 months (Fig. 4D, iii; *P*  *=* 0.015). Genotype differences in the age-related θ–low-γ PAC changes were pronounced at 6 months (Fig. 4D, iv; *P*  *<* 0.001) and between 9 and 11 months (Fig. 4D, iv; all comparisons with *P*  *<* 0.01), suggesting impairments in θ–low-γ PAC relevant to disease progression. No genotype differences were observed in age-related θ–high-γ PAC changes over the observation period (data not shown). Overall, θ–low-γ PAC thus appears to be a more sensitive indicator of cortico-hippocampal network dysfunctions in APP/PS1 mice than θ–high-γ PAC. The attenuation in cortical θ–low-γ PAC in APP/PS1 mice is evident already at 3 months and may reflect a pathophysiological change of relevance to understanding E/I changes in Alzheimer's disease.

Finally, we assessed the relationship between fE/I and θ–low-γ PAC in the PTC using repeated measures correlation analysis analogous to that applied in the HC (cf. CA1 layer-related θ and γ fE/I correlates with θ–γ PAC also during periods of age-related θ–γ PAC impairments). Similar to the results in the HC, we found that fE/I in the theta and gamma bands was positively correlated with θ–low-γ PAC over the 3- to 11-month observation period (Fig. 4E); however, the theta-band correlation was limited to a single frequency band of fE/I (6.5–8 Hz).

### Spiking activity is associated with elevated low-gamma fE/I in APP/PS1 mice

**Figure 5:**
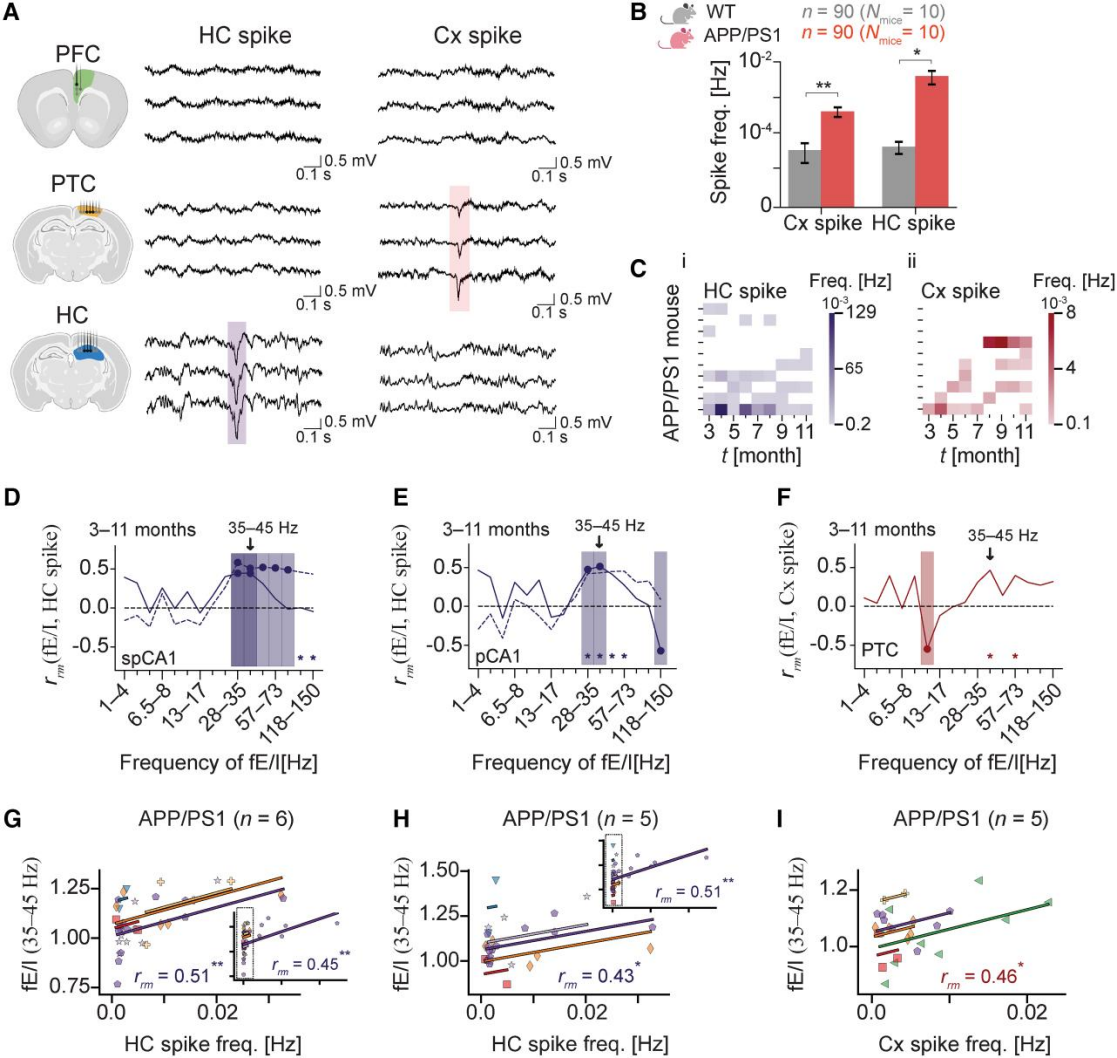
**Increased epileptiform activity in the hippocampal CA1 region of APP/PS1 mice is associated with elevated low-gamma fE/I.** (**A**) Examples of hippocampal (HC) and cortical (Cx) spiking activity observed in APP/PS1 mice. Shown are negative-polarity spikes of low amplitude recorded from PTC and HC channels, respectively. (**B**) Total average frequency of Cx and HC spikes in WT and APP/PS1 mice across 3–11 months of age. Within each month, weekly spiking frequencies were first averaged at the mouse level, and these monthly averages were then averaged at the genotype level across months. Error bars represent the standard error of the mean, and asterisks indicate significant genotype differences (Welch’s *t*-test). *N*_mice_ denotes the total number of mice per genotype, and *n* denotes the total number of data points per genotype (same for Cx and HC spikes). (**C**) Average frequency of HC (i) and Cx (ii) spikes by month (*x*-axis) for each APP/PS1 mouse (*y*-axis). Corresponding data for WT mice are shown in Supplementary Fig. 6. (**D–F**) The repeated measures correlation coefficient *r*_rm_ (*y*-axis) between fE/I and the frequency of HC spikes (**D**, **E**) and Cx spikes (**F**) across the fE/I frequency spectrum (*x*-axis). In **D** and **E**, the solid line uses all data points; the dashed line excludes data points from a single APP/PS1 mouse with extremely high HC spike activity (<0.05 cut-off). Filled circles and shaded vertical regions indicate frequency bands with significant (*P*  *<* 0.01) correlations. One asterisk marks marginal significance (0.01 ≤ *P* < 0.05). Arrows highlights the low-gamma (35–45 Hz) interval of fE/I, shown in scatterplots in **G–I**. **(G–I)** Scatterplots for weekly paired spiking frequency and low-gamma (35–45 Hz) fE/I for spCA1 (**G**), pCA1 (**H**) and PTC (**I**). The insets (**G**, **H**) show scatterplots and correlations without outlier data points, where dashed rectangles denote the data shown in the main plots (i.e. <0.05). A repeated measures correlation test was used to estimate the correlation coefficient, *r*_rm_, between fE/I and spiking frequency. Each dot represents electrode-level data for a given week and is colour- and shape-coded by mouse. Coloured lines show the best linear fit per mouse. *P  *<* 0.05; **P  *<* 0.01; ***P  *<* 0.001. WT, wild-type; APP/PS1, APPswe/PSEN1de9; PFC, prefrontal cortex; PTC, parietal cortex; HC, hippocampus; spCA1, supra-pyramidal layer of CA1; pCA1, pyramidal layer of CA1; fE/I, functional excitation–inhibition. The depicted sagittal brain views with electrode configuration in **A** were created with BioRender.com (Krivoshein, G. (2025); https://BioRender.com/33ek6gw).

Abnormal spiking activity has been reported in the APP/PS1 mice,^32^ including cortical and hippocampal spikes, SWDs and giant spikes. We examined the occurrence of these events across the PFC, PTC and HC during the LFP recording window. To account for variations in vigilance states across mice, spike counts were normalized to the total recording time and expressed as frequency (Hz). While SWDs and giant spikes appeared exclusively in APP/PS1 mice (Supplementary Figs 5A–C), cortical and hippocampal spikes also occurred in WT mice (Supplementary Fig. 6). However, both spike types were overall more frequent in APP/PS1 mice (Fig. 5A and B; *P*  *<* 0.01 for cortical spikes; 0.01 ≤ *P* < 0.05 for hippocampal spikes). Hippocampal spikes were detected in 9/10 APP/PS1 animals, with 45% of the mice exhibiting them across ages, and 1 mouse displaying an exceptionally high spike count (Fig. 5C, i). Cortical spiking was detected in 7/10 APP/PS1 mice (Fig. 5C, ii), while giant spikes and SWDs were observed in 2 and 3 APP/PS1 mice, respectively (Supplementary Fig. 5B and C). The temporal dynamics of both cortical and hippocampal isolated spikes, as represented through the distribution of ISIs, showed variable irregular patterns of spiking activity with a broad ISI range evident across different months (Supplementary Fig. 7). At 4 and 6 months, hippocampal ISI values were most prominent in the 1.4–72 s range, representing irregular spike patterns. In contrast, with older age, the hippocampal and cortical ISI distribution shifted towards shorter durations, while the average spike frequency was not increased compared to earlier ages, indicating a transition towards a more burst-like spiking pattern.

To validate fE/I as an indicator of neuronal network hyperexcitability, we correlated fE/I values at different LFP frequency ranges with epileptiform spiking activity in the cortex and HC. We used repeated measures correlation,^56^ which evaluated the within-mouse linear association between the two measures. For each mouse, weekly fE/I values in specific frequency bands were matched with the corresponding spiking frequency over the 3- to 11-month observation period. In APP/PS1 mice, hippocampal spiking frequency positively correlated with low-gamma (28–35 Hz and 35–45 Hz) fE/I in the supra-pyramidal CA1 layer (Fig. 5D), illustrated by a representative scatterplot for the 35–45 Hz band in Fig. 5G [*r*_rm_ = 0.51, 95% CI = (0.17, 0.74), *P*  *<* 0.01]. Importantly, the relationship persisted after excluding data points from the APP/PS1 mouse with exceptionally high spiking frequency [Fig. 5G, inset, *r*_rm_ = 45, 95% CI = (0.13, 0.68), *P*  *<* 0.01]. A similar, albeit outlier-sensitive, positive relationship between low-gamma fE/I and spiking frequency was observed in the pyramidal CA1 layer of APP/PS1 mice [Fig. 5E and H; *r*_rm_ = 0.43, 95% CI = (0.03, 0.71), *P*  *=* 0.036; Fig. 5H, inset, *r*_rm_ = 0.51, 95% CI = (0.19, 0.74), *P*  *<* 0.01]. No associations emerged in the infra-pyramidal CA1 layer (data not shown). Interestingly, we observed a positive, yet marginally significant, correlation between the two measures in the low-gamma (35–45 Hz) band in the PTC region of APP/PS1 mice [Fig. 5F and I; *r*_rm_ = 0.46, 95% CI = (0.05, 0.74), *P*  *=* 0.029]. Intriguingly, in the PTC, cortical spiking frequency negatively correlated with alpha fE/I (10–13 Hz) [Fig. 5F; Supplementary Fig. 8; *r*_rm_ = −0.55, 95% CI = (−0.79, −0.15), *P*  *<* 0.01], where fE/I < 1 aligned with increased cortical spiking frequency. Given their low incidence, SWD events and giant spikes were not analysed. Similarly, because WT mice provided insufficient data points (on average, only 7.7 data points per CA1 layer, corresponding to 2–3 data points per mouse; only 2 data points from a single mouse in the PTC), analyses focused on APP/PS1 mice only.

We also examined whether θ–low-γ PAC correlated with spike frequency in APP/PS1 mice. Monthly θ–low-γ PAC values were matched with monthly averaged spiking frequencies over the 3- to 11-month observation period. No correlations were observed between θ–low-γ PAC and spiking frequency in either the HC or the PTC (data not shown).

Overall, these findings indicate potential links between regional E/I imbalances and aberrant spiking activity in APP/PS1 mice. Higher low-gamma fE/I ratios (i.e. fE/I > 1) were associated with increased spiking, most pronounced in the HC, consistent with an excitation-dominant circuit that could underlie hyperexcitability. Additionally, in the PTC, lower alpha-band fE/I ratios (i.e. fE/I < 1) were linked to increased spiking, suggesting a role of inhibition-dominated circuitry in cortical hyperexcitability. Whether these E/I–spiking relationships are specific to APP/PS1 mice remains to be explored in future studies.

## Discussion

Here we investigated hippocampal and cortical E/I changes in a mouse model of Alzheimer's disease from 3 up to and including 11 months of age, using the LFP-derived fE/I ratio, θ–γ PAC and epileptiform features. In dorsal CA1, APP/PS mice showed a transient increase in low-gamma (35–45 Hz) fE/I (>1) at 6–7 months, particularly in the supra-pyramidal and pyramidal layers, with a less pronounced increase in the PTC at 6 months. Conversely, the infra-pyramidal CA1 displayed elevated low-gamma fE/I as early as 3–4 months. Additionally, APP/PS1 mice exhibited a sustained decrease in θ–γ (40–90 Hz) PAC from 6 to 11 months in CA1 and consistently lower θ–γ PAC levels in the PTC. In APP/PS1 mice, in addition to epileptiform SWDs and giant spikes, increased spiking activity was evident in the cortex and HC. Theta and gamma fE/I correlated positively with θ–γ PAC in both APP/PS1 mice and WT controls, although the exact fE/I frequency ranges of this correlation varied by brain region. Elevated low-gamma fE/I correlated with increased spiking activity in APP/PS1 mice, most prominently in CA1, whereas alpha-band fE/I < 1 correlated with increased spiking in the PTC. Together, these findings support fE/I as a promising early indicator of E/I imbalance of hippocampal and cortical neuronal networks in Alzheimer's disease.

To our knowledge, this is the first study to characterize Alzheimer's disease-related E/I alterations in mice (i) at the hippocampal–cortical network level, (ii) across a broad age range and (iii) across a wide LFP frequency spectrum. Consistent with prior reports of hippocampal hyperexcitability in Alzheimer's disease,^60-64^ APP/PS1 mice exhibited increased CA1 network excitation, as indicated by fE/I > 1, around 6–7 months. This hyperexcitability, particularly evident in the pyramidal and supra-pyramidal CA1 layers, emerged in the low-gamma (35–45 Hz) range. This frequency range is of interest because literature reports on disrupted gamma oscillations including the 40 Hz range in preclinical Alzheimer's disease models, and restoring this activity can attenuate Alzheimer's disease-related pathology and ameliorate both network and cognitive deficits.^53,54,65^ Low-gamma oscillations have been associated with synaptic activity of inhibitory somatostatin (SST) and parvalbumin (PV) interneurons.^55,66^ Reduced PV cell activity and increased excitability of excitatory pyramidal neurons in CA1 of APP/PS1 mice occur around 6 months,^31^ which coincide with Aβ plaque formation and likely contribute to the elevated fE/I at this age. Although PV cells were reported to become transiently hyperexcitable at 4 months, linked to increased levels of soluble Aβ, with yet unchanged excitability of pyramidal neurons,^31^ we did not observe a shift towards inhibitory dominance (i.e. fE/I < 1) at this earlier age. This suggests that PV cell activity alone may not determine inhibitory contributions to the hippocampal low-gamma fE/I in mice in general or in APP/PS1 mice in particular. SST interneurons, which contribute to hippocampal–cortical gamma activity^66^ and exhibit impairment associated with memory deficits in APP/PS1 mice,^67^ may also play a role. Alternatively, altered excitatory input from other areas may compensate for PV neuron hyperexcitability at 4 months, resulting in balanced fE/I. The weaker, transient low-gamma fE/I increase observed in the PTC at 6 months may reflect effects of volume-conducted increased hippocampal low-gamma activity, which is conceivable given the close physical proximity of these two brain regions, though such conduction is particularly known for theta and not low-gamma activity.^68,69^

Layer-specific fE/I changes highlight distinct pathological processes along the radial axis of CA1, where superficial layers were reported to exhibit higher amyloid plaque levels.^70,71^ In the infra-pyramidal CA1 layer––comprising the *stratum radiatum* and *stratum lacunosum moleculare*––APP/PS1 mice exhibited elevated low-gamma (35–45 Hz) fE/I as early as 3–4 months, followed by a marked increase in delta (1–4 Hz) fE/I between 4 and 8 months. As opposed to the pyramidal (*stratum pyramidale*) and supra-pyramidal (*stratum oriens*) layers, where low-gamma fE/I elevations likely reflect altered local E/I balance, the infra-pyramidal layer contains apical dendrites and serves as the main integration hub for both local inhibitory circuits^72^ and cross-regional excitatory inputs from the CA3, entorhinal cortex, and thalamus.^70,71^ The early fE/I changes in this CA1 layer may, thus, indicate disturbed external drive from the mentioned brain regions, contributing to hippocampal hyperexcitability. Given the early vulnerability of the entorhinal cortex in Alzheimer's disease––characterized by a pronounced pathology, selective impairment of neuronal inhibition and associated network hyperexcitability^71,73-75^––this region could, thereby, be a critical driver. Further investigation is needed though to clarify the cellular and network mechanisms underlying the layer- and frequency-specific fE/I changes in CA1.

Previous studies on fE/I ratios in Alzheimer's disease patients, using MEG data, have shown mixed results. Javed *et al*.^26^ reported elevated whole-brain fE/I (>1) in the 5–35 Hz range in patients with MCI due to probable Alzheimer's disease compared to healthy controls and in the 5–25 Hz range compared to individuals with subjective cognitive decline (SCD). Higher fE/I ratios also predicted subsequent conversion from MCI to Alzheimer's disease dementia. In contrast, Nifterick *et al*.^27^ found elevated cortical and hippocampal fE/I ratios (yet close to 1) in the 6–13 Hz range among patients diagnosed with probable Alzheimer's disease dementia compared to healthy elderly, with no differences in SCD or MCI groups. These inconsistencies complicate direct comparisons between mouse models and humans. Studying network activity during truly presymptomatic stages in humans is inherently difficult, as individuals may differ in their time from disease onset and not all individuals with SCD progress to Alzheimer's disease dementia.^76^ Future studies could consider investigating fE/I ratios ideally in presymptomatic individuals who carry one of the mutations that were present in the used mouse model and hence are at high risk of developing dementia to better evaluate the translational relevance of the current findings in mice. When translating preclinical findings from LFP to clinical scalp EEG, deep sources weaken and blend with cortical activity, so information from subcortical network activities will be masked, especially fine-grained intrahippocampal fE/I differences, as identified from our multisite hippocampal LFP recordings. Applying source modelling to high-density scalp EEG or MEG can help solve this issue in future clinical studies by improving spatial specificity and recovering information from subcortical signals, including those related to hippocampal activities. Additionally, given the established link between aberrant network activity in Alzheimer's disease mice and cognitive deficits,^7^ correlating preclinical E/I changes in hippocampal–cortical networks (including both fE/I and impaired θ–γ PAC) with the onset of cognitive decline could help build a clinically relevant framework for interpreting and translating the functional consequences of the preclinical E/I findings.

We also found significant impairments in θ–γ PAC, consistent with its established disruption in both Alzheimer's disease patients^38^ and mouse models.^39,41^ Since θ–γ PAC strongly reflects the functioning of inhibitory circuits involving an interplay of hippocampal local PV and longer-range connecting SST-positive oriens lacunosum moleculare interneurons,^34,47,77^ such impairment adds to growing evidence of dysfunctional inhibitory neuronal networks contributing to the early symptomatic hyperexcitability observed in Alzheimer's disease.^55,78,79^ Notably, reduced θ–γ PAC appeared earlier in the PTC where it was evident already at the 3-month window than in CA1, where a gradual decrease appeared only from 6 months onwards. Given that hippocampal θ oscillations are transmitted to the PTC via volume conduction while parietal γ oscillations are generated locally,^68^ the early θ–γ PAC deficit in the PTC likely reflects impaired communication between hippocampal θ and local cortical γ activity. Both for hippocampal and cortical LFP, the impairment of θ–γ PAC predominantly concerned the coupling of θ–low-γ (range 40–90 Hz) activity, whereas the coupling of θ–high-γ activity was unaltered in the cortex, and in supra-pyramidal CA1 was only impaired between 10 and 11 months old. In our earlier work, using a conditional *Scn1a* knockout model for epilepsy, we showed that selective impairment of θ–low-γ PAC can be explained by selective disruption of hippocampal inhibition.^47^ The observed impairment of θ–low-γ PAC in our Alzheimer's disease model thereby suggests selective impairment of hippocampal compared to cortical inhibitory neuronal activity and fits with reported reduction in hippocampal PV and SST interneuronal functioning in the context of Alzheimer's disease pathology and memory deficits.^31,67,78^

Thus, both fE/I and θ–γ PAC indicated alterations in hippocampal–cortical E/I balance during disease progression, yet they captured distinct aspects of network dysfunction. Transient and low-gamma-confined fE/I elevations contrasted with enduring and broader gamma-band θ–γ PAC impairments. This contrast may reflect that θ–γ PAC more specifically indexes the temporal coordination of activity of inhibitory circuits, while the fE/I ratio reflects the net balance between excitation and inhibition, highlighting their synergistic values as potential biomarkers. Correlation analyses revealed a positive association between θ–γ PAC and both theta- and gamma-band fE/I ratios across APP/PS1 and WT mice, suggesting that theta and gamma oscillations may jointly regulate these network features––a relationship that persisted even during the periods of age-related θ–γ PAC impairments in APP/PS1 mice. Furthermore, although no correlation was found between spiking frequency (indicative of an epileptiform phenotype in APP/PS1 mice^31,32,67,78^) and the strength of θ–γ PAC, spiking frequency correlated specifically with low-gamma fE/I levels, highlighting its potential as an early marker for symptomatic epilepsy features.^80,81^

### Strengths and limitations

A key strength of this study is its longitudinal design, spanning weekly time points between 3 and up to 12 months throughout the aging process in Alzheimer's disease mice. This approach overcomes common limitations of cross-sectional studies, narrower age windows or sparse sampling, thereby enhancing the comparability of our findings with those of other investigations. Additionally, employing Bayesian multilevel statistical modelling provided a robust framework for analysing the complex repeated measures data. Given the early exploratory nature of our study, we chose to use weakly informative priors so that our results would be driven primarily by the observed data rather than by strong assumptions. While previous studies support the presence of an E/I imbalance in both Alzheimer's disease models and patients, those findings were based on different methods of measuring E/I. In contrast to these earlier approaches, our approach estimates E/I using a novel frequency-based metric, and currently little is known about how that imbalance behaves across frequency bands or developmental stages. As the research field progresses and more is understood about these dynamics, we envisage that future research can incorporate stronger, more targeted priors to improve the precision of our inferences.

Although light-phase recordings allowed us to capture multiple vigilance states, we realize that brain activities during wakefulness in the dark phase may be different as this is the phase when rodents are usually most active. In addition, our study used only male animals and, therefore, did not account for sexual dimorphism, with the higher prevalence and rate of disease progression that have been reported for women with Alzheimer's disease.^82^ Another consideration is that the fE/I ratio was assessed during the quiet wake state, whereas θ–γ PAC was evaluated during the active wake state. Although this discrepancy in behavioural states may limit the direct comparability of the two measurements, it also underscores the relevance of tracking of excitability-relevant changes across distinct behavioural states.

Further, a more dynamic approach assessing fE/I prior to spike events would allow for claims about the value of fE/I in predicting epileptiform activity changes. However, this can only work for epileptiform activities that are not too frequent, such as SWDs or giant spikes that in our study were only infrequently recorded across the cohort of mice. For the hippocampal and cortical spikes recorded in our dataset, ISIs were typically below 60 s (in line with relatively high interictal spike frequencies, as reported also by others for the APP/PS1 model^32^), whereas fE/I requires at least 2 min of data to yield stable and reliable estimates.^21,22^ While we could not assess rapid fluctuations of fE/I in the periods immediately preceding hippocampal or cortical spikes, our approach—that is based on cleaned LFP data extracted before and after spikes—captured the overall relationship between network hyperexcitability and epileptiform activity. The data underscore that the elevated fE/I ratio reflects a network’s tendency towards hyperexcitability, given its alignment with the likelihood of spike occurrence. Lastly, to address multiple comparisons across 16 fE/I frequency bands, we applied a more stringent significance threshold of 0.01 while reporting findings with 0.01 ≤ *P* < 0.05 as potentially meaningful trends. While this minimized false positives, it did not involve a formal multiple comparison correction method, leaving some residual risk of type I error.

## Conclusions and future directions

Taken together, our study provides a comprehensive characterization of hippocampal–cortical E/I changes in a mouse model of Alzheimer's disease by integrating multiple E/I indicators across various brain regions over an extended age period, from youth to advanced age. We reveal early, frequency-specific shifts towards excitatory dominance signalled by elevated fE/I ratios and uncover associations between fE/I, θ–γ PAC and epileptiform activity. Our findings support further validation of fE/I as a promising EEG-based indicator for therapeutic monitoring of Alzheimer's disease features that are related to underlying changes in brain E/I balance. Prior experimental studies have shown that restoring interneuron function can ameliorate network hyperexcitability and improve cognitive performance in Alzheimer's disease mouse models,^31,65^ underscoring the potential of targeting E/I balance as a treatment strategy. Importantly, fE/I can be computed from recordings as short as 2 min^21,22^ and does not depend on the presence of epileptiform activity, which makes it suitable for routine clinical assessments. Our preclinical data underscore that fE/I is a metric that is sensitive to neurophysiological alterations related to hyperexcitability that are relevant to disease progression in Alzheimer's disease, i.e. epileptiform activity and impairments in hippocampal–cortical θ–γ PAC that are indicative of cortical decline. Since E/I imbalance is not unique to Alzheimer's disease and can be observed in other neurological conditions, changes in fE/I should not be interpreted as Alzheimer's disease-specific and thereby fE/I features alone may not have direct diagnostic value. Also, since fE/I captures the net effect on E/I balance from many interacting mechanisms,^21^ different interactions can, in principle, produce similar net E/I readouts. Moreover, without a normative distribution, it is impossible to discriminate pathological E/I shifts from normal variation. Given the translatability to clinical EEG, however, fE/I has future clinical potential as an early indicator of hyperexcitability changes associated with epileptiform features and cognitive decline in people with Alzheimer's disease. As such, it could, alone or in combination with other biomarkers, help indicate disease progression or assess effects of treatments affecting E/I-related disease features.

While our current assessment of fE/I was focused on the quiet awake state to align with typical resting-state EEG paradigms in humans, future studies could extend these measurements to other vigilance states, such as rapid-eye-movement and non-rapid-eye-movement sleep. After all, sleep is critical for memory consolidation^83^ and Aβ clearance^84^ and is often disrupted in Alzheimer's disease mouse models and patients.^29^ Additionally, evaluating fE/I during cognitive challenges could help clarify how early network imbalances contribute to emerging cognitive deficits in Alzheimer's disease.

## Supplementary Material

fcaf443_Supplementary_Data

## Data Availability

All data are available upon request to R.E.v.K. The analysis code for fE/I and θ–γ PAC can be found at https://doi.org/10.6084/m9.figshare.29715806. The standalone Python implementation of the fE/I algorithm is available under a CC-BY-NC-SA licence at https://github.com/arthur-ervin/crosci.
