# Supplementary material for: Hippocampal and cortical activities reflect early hyperexcitability in an Alzheimer's mouse model
Source: Brain Commun. 2025 Nov 12;7(6):fcaf443. doi: 10.1093/braincomms/fcaf443 (PMC12641122; doi:10.1093/braincomms/fcaf443)
Supplement: fcaf443_Supplementary_Data [file fcaf443_supplementary_data.zip › Supplementary Figures.docx]

**
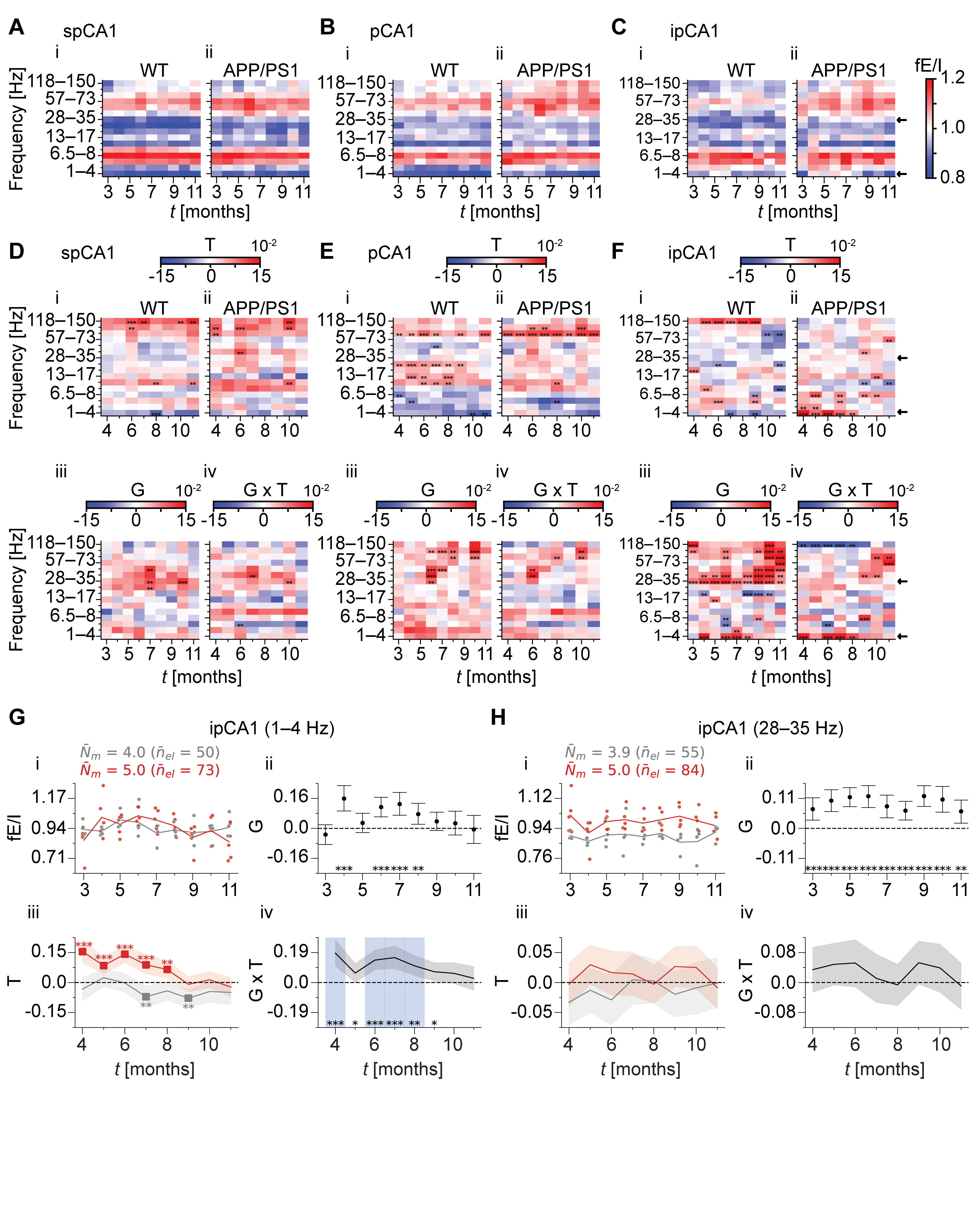
**

**Supplementary Figure 1 fE/I indicates reduced inhibition in hippocampal CA1 of APP/PS1 mice.**

**(A, B, C)** Age-related, genotype-averaged fE/I across 1–150 Hz spectrum (y-axis) for hippocampal spCA1 **(A)**, pCA1 **(B)**, and ipCA1 **(C)**. Data are shown from 3 to 11 months of age (x-axis). fE/I values were first averaged at the mouse-electrode level across weeks for a given month and then at the mouse level across electrodes per month. Plots (i) and (ii) represent the WT and APP/PS1 genotype, respectively. **(D, E, F)** BMLM was used to estimate the age effect for WT (i) and APP/PS1 (ii) mice, the genotype effect (iii), and their interaction (iv) across the fE/I frequency bands for spCA1 (D), pCA1 (E), and ipCA1 (F). Color represents posterior mean parameter estimate, and asterisks mark significant effects (here, only *P <* 0.01 are shown). For all effects, their 95% confidence intervals (CI95) and *P*-values, as well as the total number of analyzed data points per frequency band, can be found in Excel tables in Supplementary Files 1, 2, and 3 for spCA1, pCA1, and ipCA1, respectively. **(G, H)** Early fE/I changes in APP/PS1 mice detected for delta (1–4 Hz in G) and borderline beta-low-gamma (28–35 Hz in H) oscillations in ipCA1. (i) Data points represent individual mice and are shown for each genotype (grey: WT, red: APP/PS1) at each month, with lines representing the corresponding genotype means. Each data point at each month reflects a mouse’s fE/I value, first averaged across weeks at the mouse-electrode level, then averaged across electrodes at the mouse level. $\overline{n}$_el_ and $\overline{N}$_m_ denote the average number of total electrodes and mice with computed fE/I per genotype across months, respectively. BMLM was used to estimate (ii) the genotype effect, (iii) the age effect, and (iv) their interaction. Dots in (ii) and lines in (iii, iv) represent posterior mean parameter estimates. In total, 1184 data points were analyzed in G and 1337 in H. Error bars in (ii), grey (for WT) and red (for APP/PS1) shaded areas in (iii), and dark shaded area in (iv) indicate 95% confidence intervals. Time points with significant differences are indicated by asterisks in (ii), filled squares and asterisks colored by genotype in (iii), and by light blue regions and asterisks in (iv). *: *P <* 0.05; **: *P <* 0.01; *** *P <* 0.001. WT: wildtype; APP/PS1: APPswe/PSEN1de9; spCA1: supra-pyramidal layer of CA1; pCA1: pyramidal layer of CA1; ipCA1: infra-pyramidal layer of CA1; BMLM: Bayesian multilevel modeling; G: genotype effect; T: age effect; G x T: interaction effect between genotype and age; LFP: local field potential; fE/I: functional excitation-inhibition ratio.

**
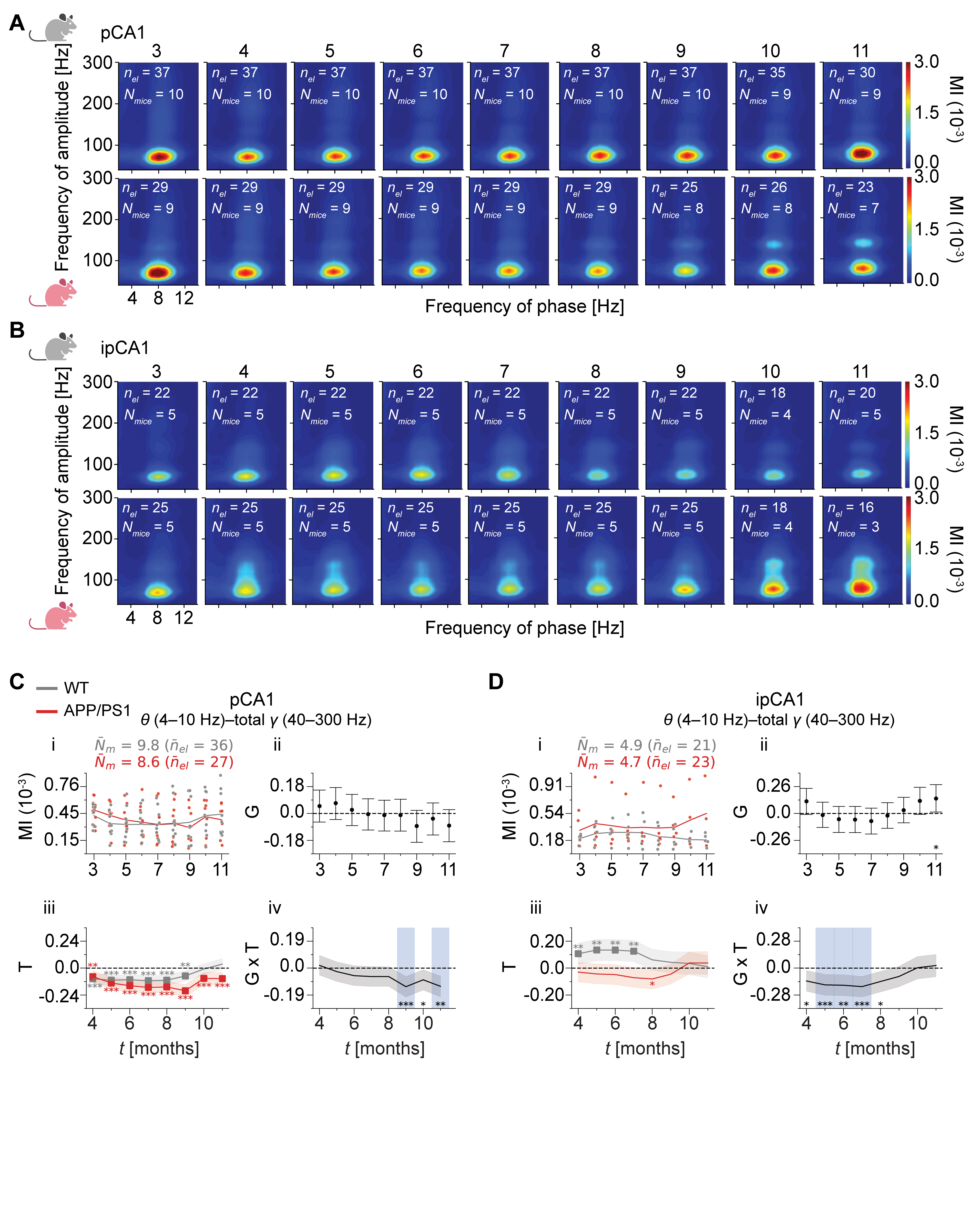
**

**Supplementary Figure 2 Modulation in the θ-high-γ frequency range is not consistently observed in hippocampal pCA1 and ipCA1.**

**(A, B)** Genotype -averaged θ-γ PAC comodulograms of hippocampal (pCA1 in A, and ipCA1 in B) LFP in WT (top row) and APP/PS1 mice (bottom row) during active wakefulness from 3 and 11 months of age (columns). All subplots share the same x-axis and y-axis. Corresponding data for the supra-pyramidal CA1 (spCA1) layer are shown in Figure 3 of the main text. **(C, D)** Age-related impairments in θ-total-γ PAC in pCA1 **(C)** and ipCA1 **(D)** are less prominent than those observed in θ-low-γ PAC in Fig. 3C and 3D. (i) Age-related θ-total-γ PAC. Data points represent individual mice and are shown for each genotype (grey: WT, red: APP/PS1) at each month, with lines representing the corresponding genotype means. Each data point at each month reflects a mouse’s θ-total-γ PAC value, averaged across electrodes at the mouse level per month. $\overline{n}$_el_ and $\overline{N}$_m_ denote the average number of total electrodes and mice with computed θ-total-γ PAC per genotype across months, respectively. BMLM was used to estimate the genotype effect (ii), age effect (iii), and their interaction (iv). Dots in (ii) and lines in (iii, iv) represent posterior mean parameter estimates. In total, 572 data points were analyzed in pCA1 and 401 in ipCA1. Error bars in (ii), grey (for WT) and red (for APP/PS1) shaded areas in (iii), and dark shaded area in (iv) indicate 95% confidence intervals. Time points with significant differences are indicated by asterisks in (ii), filled squares and asterisks colored by genotype in (iii), and by light blue regions and asterisks in (iv). *: *P <* 0.05; **: *P <* 0.01; *** *P <* 0.001. WT: wildtype; APP/PS1: APPswe/PSEN1de9; pCA1: pyramidal layer of CA1; ipCA1: infra-pyramidal layer of CA1; θ-γ PAC: theta-gamma phase-amplitude coupling; BMLM: Bayesian multilevel modeling; G: genotype effect; T: age effect; G x T: interaction effect between genotype and age; LFP: local field potential; MI: modulation index (see Materials and Methods, θ-γ PAC analysis section).

**
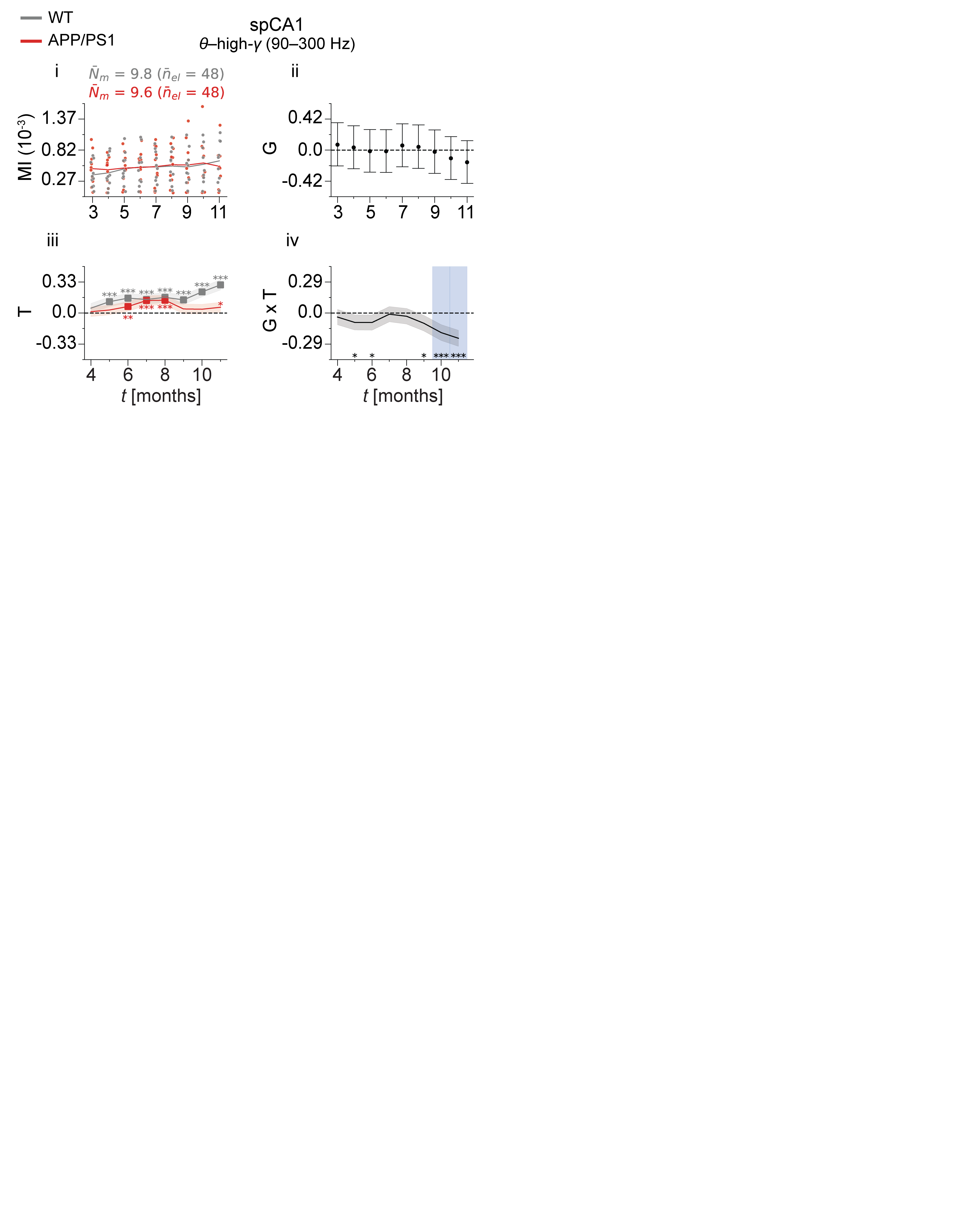
**

**Supplementary Figure 3 Age-related θ-high-γ PAC impairment in hippocampal CA1 of 10-to-11-month-old APP/PS1 mice.**

Age-related changes in θ-high-γ PAC for LFP in hippocampal spCA1. (i) Data points represent individual mice and are shown for each genotype (grey: WT, red: APP/PS1) at each month, with lines representing the corresponding genotype means. Each data point at each month reflects a mouse’s θ-high-γ PAC value, averaged across electrodes at the mouse level per month. $\overline{n}$_el_ and $\overline{N}$_m_ denote the average number of total electrodes and mice with computed θ-high-γ PAC across months, respectively. BMLM was used to estimate the genotype effect (ii), age effect (iii), and their interaction (iv). Dots in (ii) and lines in (iii, iv) represent posterior mean parameter estimates. In total 874 data points were analyzed. Error bars in (ii), grey (for WT) and red (for APP/PS1) shaded areas in (iii), and dark shaded area in (iv) indicate 95% confidence intervals. Time points with significant differences are indicated by asterisks in (ii), filled squares and asterisks colored by genotype in (iii), and by light blue regions and asterisks in (iv). *: *P <* 0.05; **: *P <* 0.01; *** *P <* 0.001. WT: wildtype; APP/PS1: APPswe/PSEN1de9; spCA1: supra-pyramidal layer of CA1; θ-γ PAC: theta-gamma phase-amplitude coupling; BMLM: Bayesian multilevel modeling; G: genotype effect; T: age effect; G x T: interaction effect between genotype and age; LFP: local field potential; MI: modulation index (see Materials and Methods, θ-γ PAC analysis section).

**
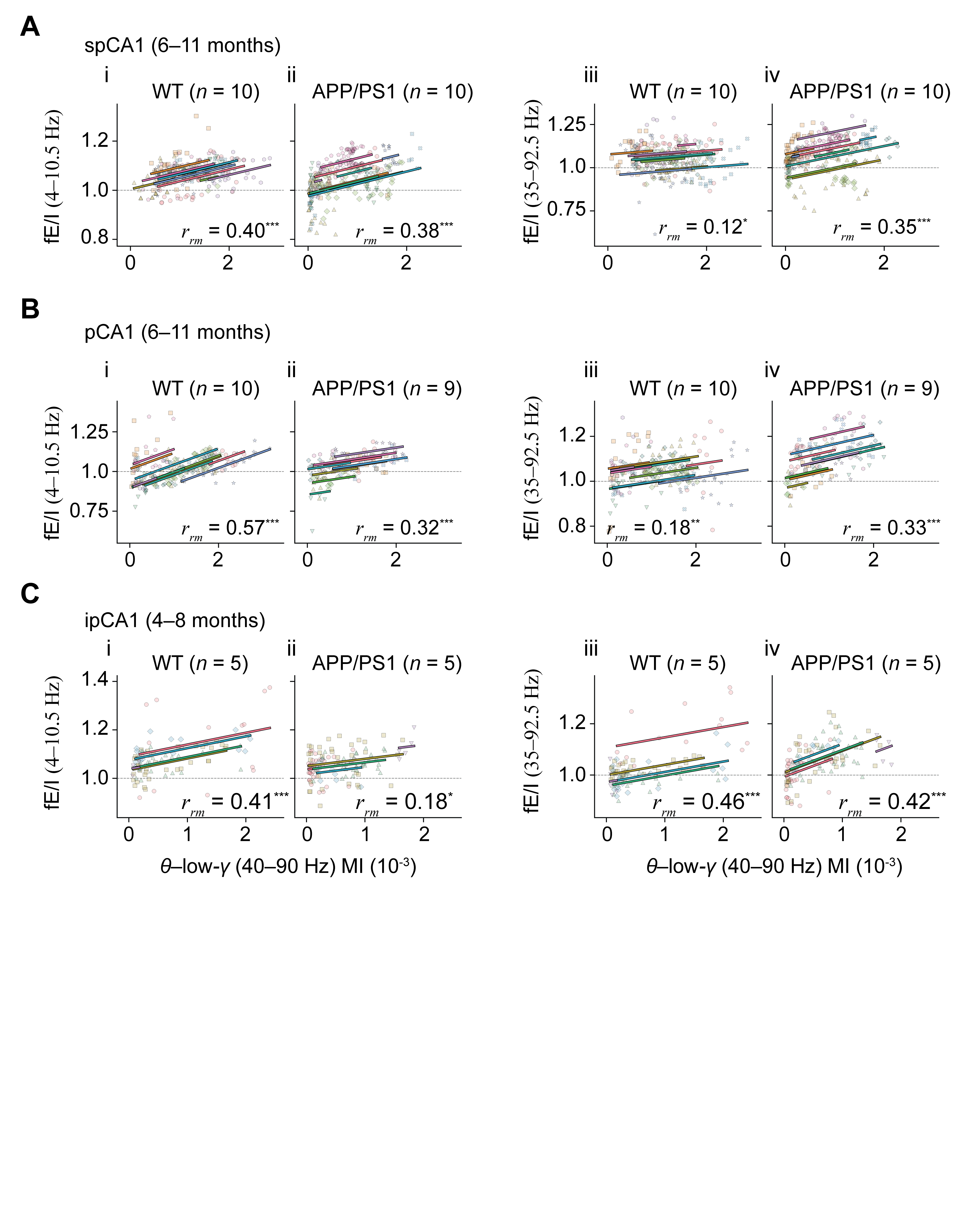
**

**Supplementary Figure 4 The relationship between fE/I and θ-γ PAC is maintained during periods of θ-γ PAC impairment in hippocampal CA1 of APP/PS1 mice.**

**(A, B, C)** Scatterplots for monthly paired θ-low-γ PAC and fE/I in hippocampal spCA1 **(A)**, pCA1 **(B)**, and ipCA1 **(C)**. (i, ii) θ-low-γ PAC vs. average fE/I for the theta band (bins in the 4–10.5 Hz range) for WT (i) and APP/PS1 (ii) mice during the age windows of age-related θ-low-γ PAC impairment in APP/PS1 mice. (iii, iv) θ-low-γ PAC vs. average fE/I for the gamma band (bins in the 35–92.5 Hz range) for WT (iii) and APP/PS1 (iv) mice. A repeated measures correlation test was used to estimate the correlation coefficient, $r_{rm}$, between fE/I and θ-low-γ PAC. Each dot represents electrode-level data for a given month and is colored and shaped my mouse. Colored lines show the best linear fit per mouse. *: *P <* 0.05; **: *P <* 0.01; *** *P <* 0.001. WT: wildtype; APP/PS1: APPswe/PSEN1de9; spCA1: supra-pyramidal layer of CA1; pCA1: pyramidal layer of CA1; ipCA1: infra-pyramidal layer of CA1; θ-γ PAC: theta-gamma phase-amplitude coupling; fE/I: functional excitation-inhibition ratio.

**
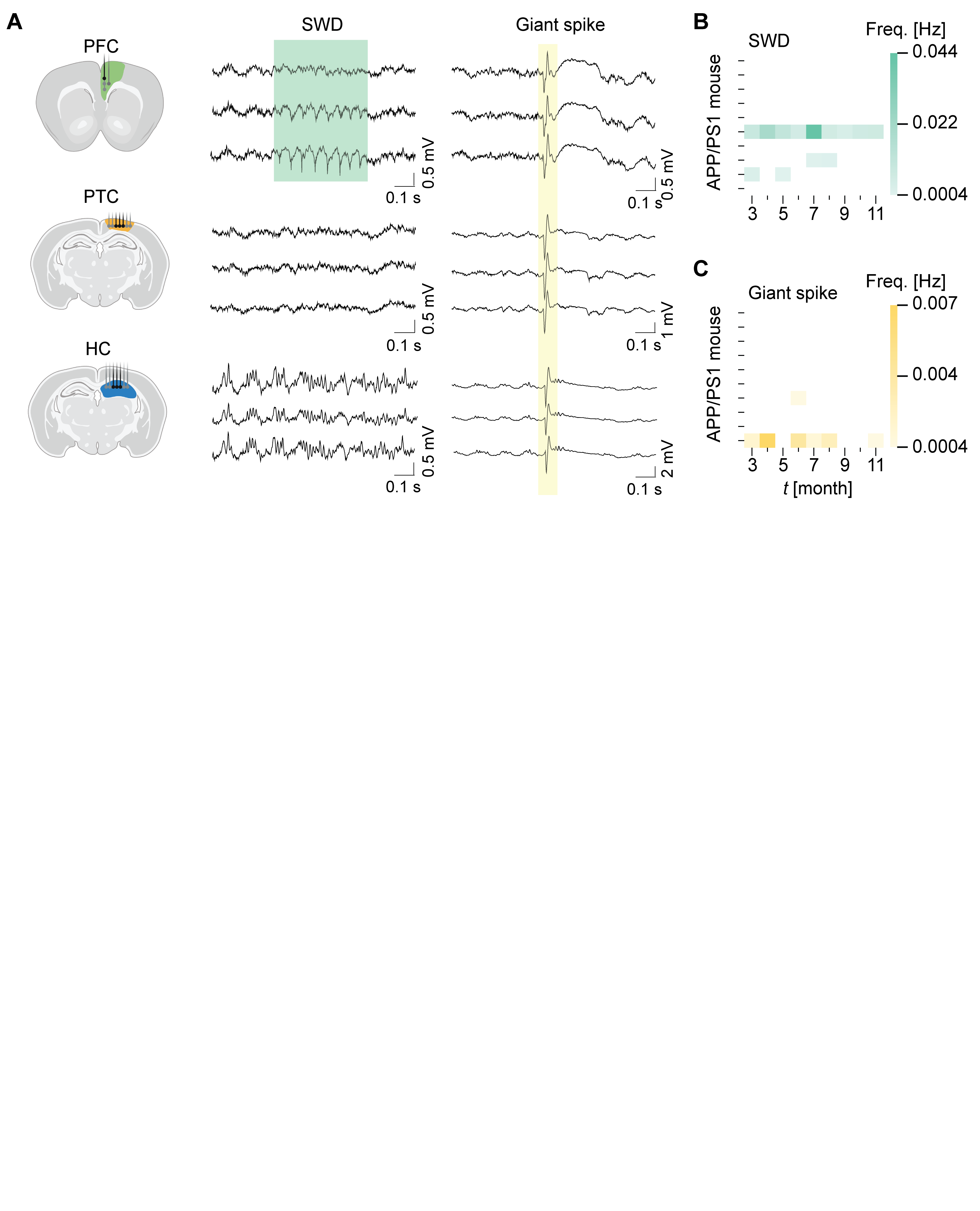
**

**Supplementary Figure 5 SWD and giant spikes occur in APP/PS1 mice.**

**(A)** Examples of spike wave discharges (SWD) and giant spikes that are infrequently observed in APP/PS1 mice. Giant spikes are present across all channels and brain regions, consisting of two components (a negative and a positive spike) followed by a ~200-ms hyperpolarization shift in HC channels. SWD appear as a repeating, regular pattern in PFC channels, without any synchronous activity in HC or PTC channels. **(B, C)** Frequency of SWD **(B)** and giant spikes **(C)** at each month (x-axis) for each APP/PS1 mouse (y-axis), averaged per mouse across all recordings for within that month. APP/PS1: APPswe/PSEN1de9; PFC: prefrontal cortex; PTC: parietal cortex; HC: hippocampus. The depicted sagittal brain views with electrode configuration in (A) were created in BioRender. Krivoshein, G. (2025) <https://BioRender.com/eem133x>.

**
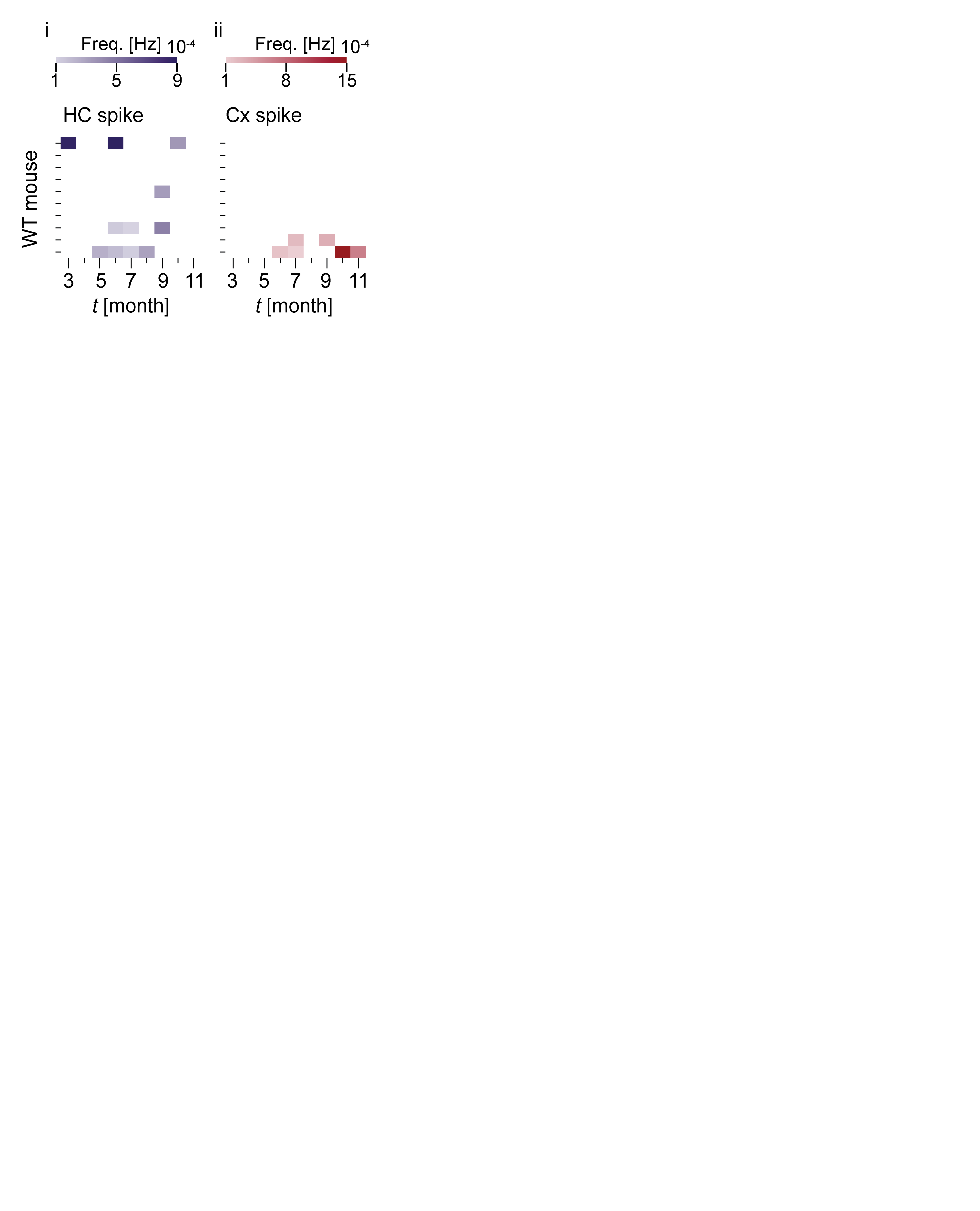
**

**Supplementary Figure 6 Hippocampal and cortical spikes occur in WT mice.**

Frequency of hippocampal (HC) (i) and cortical (Cx) (ii) spikes at each month (x-axis) for each wildtype (WT) mouse (y-axis), averaged per mouse across all recordings within that month. Note that hippocampal and cortical spiking frequency in WT mice is much lower than that observed in APP/PS1 mice (see main Fig. 5). APP/PS1: APPswe/PSEN1de9.


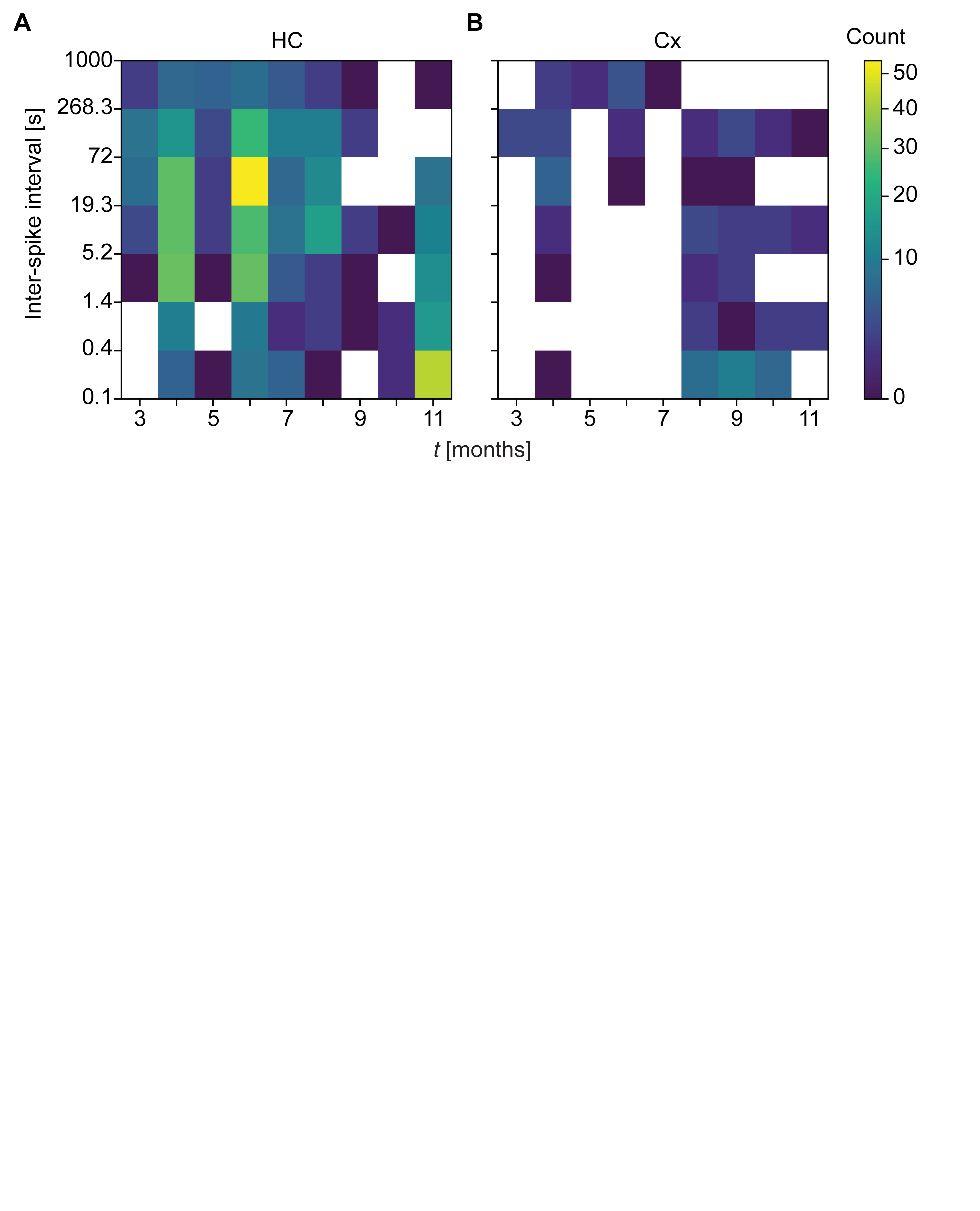


**Supplementary Figure 7 Distribution of inter-spike intervals (ISIs) of LFP spikes in APP/PS1 mice.**

ISIs were computed from LFP recorded in hippocampus (HC) **(A)** and cortex (Cx) **(B)** across 3 to 11 months of age in APP/PS1 mice. The color coding indicates the number of ISIs encountered per recording period. Note that at 4 and 6 months, hippocampal ISI values were most prominent in the 1.4–72 s range, representing irregular spike patterns. In contrast, with older age, the hippocampal and cortical ISI distribution shifted toward shorter durations, while the average frequency was not increased compared to earlier ages, indicating a transition toward a more burst-like spiking pattern. APP/PS1: APPswe/PSEN1de9.

**
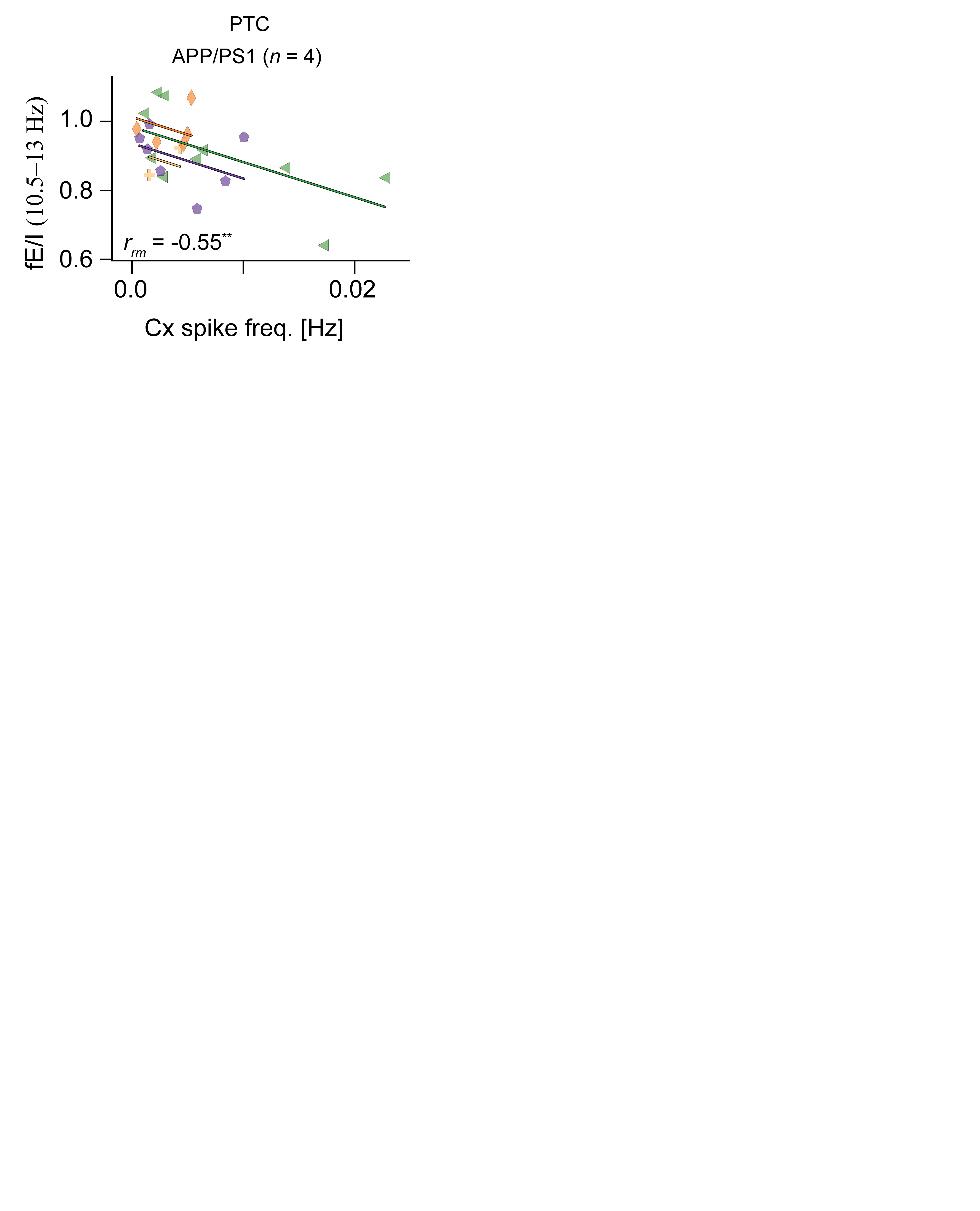
**

**Supplementary Figure 8 Inhibition-dominated alpha fE/I is associated with increased cortical spiking in APP/PS1 mice.**

Scatterplot for weekly paired cortical (Cx) spiking frequency and alpha fE/I (10.5–13 Hz). A repeated measures correlation test was used to estimate the correlation coefficient, $r_{rm}$, between fE/I and spiking frequency. Each dot represents electrode-level data for a given week and is color- and shape-coded by mouse. Colored lines show the best linear fit per mouse. **: *P <* 0.01; APP/PS1: APPswe/PSEN1de9; PTC: parietal cortex; fE/I: functional excitation-inhibition ratio.
