# Supplementary material for: Hippocampal and cortical activities reflect early hyperexcitability in an Alzheimer's mouse model
Source: Brain Commun. 2025 Nov 12;7(6):fcaf443. doi: 10.1093/braincomms/fcaf443 (PMC12641122; doi:10.1093/braincomms/fcaf443)
Supplement: fcaf443_Supplementary_Data [file fcaf443_supplementary_data.zip › Supplementary Table Legends.docx]

**Supplementary Table 1 Bayesian multilevel model estimates of genotype, age, and interaction effects on fE/I in the supra-pyramidal CA1 layer.**

Each sheet contains Bayesian multilevel model estimates of genotype, age, and interaction effects on the functional excitation-inhibition ratio (fE/I) in the supra-pyramidal CA1 layer of the dorsal hippocampus for one of the 16 frequency bands. The frequency range is specified in the sheet name. For each effect, the table reports the posterior mean coefficient, 95% credible interval, and *P*-value. The total number of analyzed data points is indicated at the bottom of each table. Abbreviations and column descriptions: WT, wildtype mice; APPPS1, APPswe/PSEN1de9 mice; G coefficient, posterior mean coefficient for the effect of genotype; G CI95.lower and G CI95.upper, lower and upper bounds of the 95% credible interval for the genotype effect; G pvalue, *P*-value for the genotype effect; T coefficient (WT), posterior mean coefficient for the age effect in WT mice; T CI95.lower (WT) and T CI95.upper (WT), lower and upper bounds of the credible interval for the age effect in WT; T pvalue (WT), *P*-value for age effect in WT; T coefficient (APPPS1), posterior mean coefficient for the effect of age in APPPS1 mice; T CI95.lower (APPPS1) and T CI95.upper (APPPS1), lower and upper bounds of the credible interval for the age effect in APPPS1; T pvalue (APPPS1), *P*-value for the age effect in APPPS1; GxT coefficient, posterior mean coefficient for the genotype x age interaction; GxT CI95.lower and GxT CI95.upper, lower and upper bounds of the credible interval for the interaction effect; GxT pvalue, *P*-value for the interaction effect; Total N, total number of analyzed data points.

**Supplementary Table 2 Bayesian multilevel model estimates of genotype, age, and interaction effects on fE/I in the pyramidal CA1 layer.**

Each sheet contains Bayesian multilevel model estimates of genotype, age, and interaction effects on the functional excitation-inhibition ratio (fE/I) in the pyramidal CA1 layer of the dorsal hippocampus for one of the 16 frequency bands. The frequency range is specified in the sheet name. For each effect, the table reports the posterior mean coefficient, 95% credible interval, and *P*-value. The total number of analyzed data points is indicated at the bottom of each table. Abbreviations and column descriptions: WT, wildtype mice; APPPS1, APPswe/PSEN1de9 mice; G coefficient, posterior mean coefficient for the effect of genotype; G CI95.lower and G CI95.upper, lower and upper bounds of the 95% credible interval for the genotype effect; G pvalue, *P*-value for the genotype effect; T coefficient (WT), posterior mean coefficient for the age effect in WT mice; T CI95.lower (WT) and T CI95.upper (WT), lower and upper bounds of the credible interval for the age effect in WT; T pvalue (WT), *P*-value for age effect in WT; T coefficient (APPPS1), posterior mean coefficient for the effect of age in APPPS1 mice; T CI95.lower (APPPS1) and T CI95.upper (APPPS1), lower and upper bounds of the credible interval for the age effect in APPPS1; T pvalue (APPPS1), *P*-value for the age effect in APPPS1; GxT coefficient, posterior mean coefficient for the genotype x age interaction; GxT CI95.lower and GxT CI95.upper, lower and upper bounds of the credible interval for the interaction effect; GxT pvalue, *P*-value for the interaction effect; Total N, total number of analyzed data points.

**Supplementary Table 3 Bayesian multilevel model estimates of genotype, age, and interaction effects on fE/I in the infra-pyramidal CA1 layer.**

Each sheet contains Bayesian multilevel model estimates of genotype, age, and interaction effects on the functional excitation-inhibition ratio (fE/I) in the infra-pyramidal CA1 layer of the dorsal hippocampus for one of the 16 frequency bands. The frequency range is specified in the sheet name. For each effect, the table reports the posterior mean coefficient, 95% credible interval, and *P*-value. The total number of analyzed data points is indicated at the bottom of each table. Abbreviations and column descriptions: WT, wildtype mice; APPPS1, APPswe/PSEN1de9 mice; G coefficient, posterior mean coefficient for the effect of genotype; G CI95.lower and G CI95.upper, lower and upper bounds of the 95% credible interval for the genotype effect; G pvalue, *P*-value for the genotype effect; T coefficient (WT), posterior mean coefficient for the age effect in WT mice; T CI95.lower (WT) and T CI95.upper (WT), lower and upper bounds of the credible interval for the age effect in WT; T pvalue (WT), *P*-value for age effect in WT; T coefficient (APPPS1), posterior mean coefficient for the effect of age in APPPS1 mice; T CI95.lower (APPPS1) and T CI95.upper (APPPS1), lower and upper bounds of the credible interval for the age effect in APPPS1; T pvalue (APPPS1), *P*-value for the age effect in APPPS1; GxT coefficient, posterior mean coefficient for the genotype x age interaction; GxT CI95.lower and GxT CI95.upper, lower and upper bounds of the credible interval for the interaction effect; GxT pvalue, *P*-value for the interaction effect; Total N, total number of analyzed data points.

**Supplementary Table 4 Bayesian multilevel model estimates of genotype, age, and interaction effects on fE/I in the parietal cortex.**

Each sheet contains Bayesian multilevel model estimates of genotype, age, and interaction effects on the functional excitation-inhibition ratio (fE/I) in the parietal cortex for one of the 16 frequency bands. The frequency range is specified in the sheet name. For each effect, the table reports the posterior mean coefficient, 95% credible interval, and *P*-value. The total number of analyzed data points is indicated at the bottom of each table. Abbreviations and column descriptions: WT, wildtype mice; APPPS1, APPswe/PSEN1de9 mice; G coefficient, posterior mean coefficient for the effect of genotype; G CI95.lower and G CI95.upper, lower and upper bounds of the 95% credible interval for the genotype effect; G pvalue, *P*-value for the genotype effect; T coefficient (WT), posterior mean coefficient for the age effect in WT mice; T CI95.lower (WT) and T CI95.upper (WT), lower and upper bounds of the credible interval for the age effect in WT; T pvalue (WT), *P*-value for age effect in WT; T coefficient (APPPS1), posterior mean coefficient for the effect of age in APPPS1 mice; T CI95.lower (APPPS1) and T CI95.upper (APPPS1), lower and upper bounds of the credible interval for the age effect in APPPS1; T pvalue (APPPS1), *P*-value for the age effect in APPPS1; GxT coefficient, posterior mean coefficient for the genotype x age interaction; GxT CI95.lower and GxT CI95.upper, lower and upper bounds of the credible interval for the interaction effect; GxT pvalue, *P*-value for the interaction effect; Total N, total number of analyzed data points.
